# Elucidating mechanistic insights into drug action for atopic dermatitis: a systems biology approach

**DOI:** 10.1186/s12895-018-0070-4

**Published:** 2018-02-07

**Authors:** Indhupriya Subramanian, Vivek K. Singh, Abhay Jere

**Affiliations:** LABS, Persistent Systems Limited, 9A/12, Erandwane, Pune, Maharashtra 411004 India

**Keywords:** Atopic dermatitis, Computational approach, Transcriptomic data analysis

## Abstract

**Background:**

Topical Betamethasone (BM) and Pimecrolimus (PC) are widely used drugs in the treatment of atopic dermatitis (AD). Though the biomolecules and biological pathways affected by the drugs are known, the causal inter-relationships among these pathways in the context of skin is not available. We aim to derive this insight by using transcriptomic data of AD skin samples treated with BM and PC using systems biology approach.

**Methods:**

Transcriptomic datasets of 10 AD patients treated with Betamethasone and Pimecrolimus were obtained from GEO datasets. We used a novel computational platform, eSkIN (www.persistent.com/eskin), to perform pathway enrichment analysis for the given datasets. eSkIN consists of 35 skin specific pathways, thus allowing skin-centric analysis of transcriptomic data. Fisher’s exact test was used to compute the significance of the pathway enrichment. The enriched pathways were further analyzed to gain mechanistic insights into the action of these drugs.

**Results:**

Our analysis highlighted the molecular details of the mechanism of action of the drugs and corroborated the known facts about these drugs i.e. BM is more effective in triggering anti-inflammatory response but also causes more adverse effect on skin barrier than PC. In particular, eSkIN helped enunciate the biological pathways activated by these drugs to trigger anti-inflammatory response and its effect on skin barrier. BM suppresses pathways like TNF and TLRs, thus inhibiting NF-κB while PC targets inflammatory genes like IL13 and IL6 via known calcineurin-NFAT pathway. Furthermore, we show that the reduced skin barrier function by BM is due to the suppression of activators like AP1 transcription factors, CEBPs.

**Conclusion:**

We thus demonstrate the detailed mechanistic insight into drug action of AD using a novel computational approach.

**Electronic supplementary material:**

The online version of this article (10.1186/s12895-018-0070-4) contains supplementary material, which is available to authorized users.

## Background

Atopic dermatitis (AD) is one of the most common disorders of skin that affects approximately 20% of children and 3% adults worldwide [1]. The pathophysiology of AD includes breakdown of the skin barrier, which in turn, initiates immunological response and inflammation [1]. The current treatment for AD involves topical application of corticosteroids or calcineurin inhibitors [2]. Betamethasone valerate (BM) and Pimecrolimus (PC) are two of the most commonly used drugs for the treatment of atopic dermatitis.

BM, a corticosteroid, is known to suppress the inflammation, but fails to adequately restore the damaged skin barrier which subsequently leads to secondary skin infections. BM binds to its corticosteroid receptor in skin and perturbs various biomolecules in keratinocytes involved in processes like inflammation, keratinocyte differentiation, proliferation and cellular adhesion [3]. On the other hand, PC, a topical calcineurin inhibitor (TCI), causes mild suppression of inflammation, but is more efficient in restoring the skin barrier. PC is known to mediate its action through NFAT signaling pathways [2].

US FDA issues TCI drugs with a boxed warning owing to a potential risk of malignancy, in spite of various studies disproving any such association [4]. This favors the use of topical corticosteroids as an alternate treatment for AD, even though it suffers from impaired skin barrier and risk of secondary skin infections as side effects. This raises the need to have a complete mechanistic insight into the action of these drugs, which can then be used to understand the factors responsible for side effects and develop better treatment for AD.

A central piece for gaining mechanistic insight into drug action is to understand the biomolecular interactions and pathways that are impacted by the drug, which in turn, determine the therapeutic efficiency and adverse effects. In this study, we have used eSkIN, a novel systems biology based computational platform specially designed to aid skin omics research. eSkIN contains a comprehensive model of skin with 35 manually-curated skin-specific pathways and 2600+ genes. This allows skin-centric analysis and interpretation of omics data, which to the best of our knowledge, is not available in other commonly used software applications (e.g. *DAVID* [5, 6] and GSEA [7]).

We present the detailed mechanistic analysis of BM and PC highlighting the biomolecular interactions and pathways involved in their mechanism of action and adverse effects. Publicly available transcriptomic data from patients treated with BM and PC [2] were used for this study and the data were analyzed using eSkIN platform. We report that distinct pathways are affected by these drugs to bring about their therapeutic effect, and we also further elucidate the importance of these pathways in the context of skin physiology.

## Methods

### Transcriptomic data

Transcriptomic data from lesional AD skin samples of 10 patients, before and after topical treatment of BM and PC twice daily for three weeks, were used in this study [2]. The data was downloaded from NCBI GEO (Gene Expression Omnibus) database using following accession number: GSE32473.

### Normalization and quality check

The datasets of BM and PC were analyzed separately. All the samples were normalized as per Jensen et al., [2]. Briefly, each sample was normalized with 50^th^ percentile (median) of that sample. To ensure quality of the input data, only probe sets with present or marginal calls in at least 70% of samples per analysis group were considered. Median expression values of probes were assigned to gene.

### Data analysis

eSkIN (www.persistent.com/eskin) was used to perform skin-centric analysis of the transcriptomic data owing to the availability of 35 manually-curated skin-specific pathways (Additional file 1: Table S1) and 2600+ genes in this platform. The 35 pathways represent following functional categories of skin physiology: Basic skin physiology, Epidermal formation, Pigmentation and Stress response. The eSkIN pathways include molecular interactions that detail the roles played by various biomolecules (e.g. genes, proteins and small molecules) in a particular pathway.

We computed the Log_2_ fold change of the genes with respect to baseline (before topical treatment) samples of respective drug. Two fold change was used as a threshold to identify differentially expressed genes i.e. up-regulated genes ≥ + 1 Log_2_ fold change and down-regulated genes ≤ − 1 Log_2_ fold change. Sensitivity analysis of fold change cutoff was performed by increasing and decreasing one fold change of the default value to understand its effect on our analysis (see Additional files 2, 3 and 4). As observed, the key pathways contributing towards the drug action are captured by all the three fold change cutoffs. Hence, for further analysis we used the default cutoff (Log_2_ fold change = 1).

#### Pathway enrichment analysis using eSkIN

Pathway enrichment analysis is based on the assumption that behavior of the genes involved in the same biological pathways is correlated. Using statistical methods, it helps to identify the most perturbed pathways based on an input set of genes [8]. Such analysis is widely used to gain insight into functional roles of differentially expressed genes [9, 10]. Statistical methods like Fisher’s test, hypergeometric, binomial, bayesian and chi-squared are widely used in pathway enrichment analysis [6].

We used the skin-centric knowledge-base of eSkIN as the backend database for performing pathway enrichment analysis. This facilitates the identification of skin-specific pathways that are perturbed due to the treatment and thus, helps in understanding the skin-centric effects of the treatment. eSkIN uses Fisher’s exact test for computing the significance of the enrichment of pathways. Fisher’s exact test with following parameters is used for computing *p*-value.

1 $$ \mathrm{p}=\frac{\left(\begin{array}{c}\mathrm{a}+\mathrm{b}\\ {}\mathrm{a}\end{array}\right){\left(\begin{array}{c}\mathrm{c}+\mathrm{d}\\ {}\mathrm{c}\end{array}\right)}^{`}}{\left(\begin{array}{c}\mathrm{n}\\ {}\mathrm{a}+\mathrm{c}\end{array}\right)}=\frac{\left(\mathrm{a}+\mathrm{b}\right)!\left(\mathrm{c}+\mathrm{d}\right)!\left(\mathrm{a}+\mathrm{c}\right)!\left(\mathrm{b}+\mathrm{d}\right)!}{\mathrm{a}!\mathrm{b}!\mathrm{c}!\mathrm{d}!\mathrm{n}!} $$

In Eq. (1), a = number of unique differentially expressed genes (DEGs) in a pathway in eSkIN knowledge-base, b = number of unique DEGs in eSkIN knowledge-base excluding DEGs in that pathway, c = number of unique non-DEGs in that pathway, d = number of unique genes in eSkIN knowledge-base that are non-DEG and not part of that pathway, n = a + b + c + d, and $\left(\genfrac{}{}{0pt}{}{\mathrm{n}}{\mathrm{k}}\right)$ represents binomial coefficient. The *p*-value from Fisher’s exact test is a measure of the chance of random association between differentially expressed genes and a pathway. Smaller the p-value, lower is the random chance, and thus, higher is the likelihood that a pathway is significantly enriched.

Furthermore, eSkIN eliminates the need to average out sample level information as it allows analysis of multiple samples simultaneously. The enriched pathways (i.e. eSkIN *p*-value < 0.05) in BM and PC treated samples were further analyzed by overlaying the transcriptomic data on these pathways using Gene Expression Overlay feature of eSkIN. This feature of eSkIN allows pathway enrichment analysis and visualization of transcriptomic data in the context of skin related pathways. The genes are colored based on their expression levels, and thus, helps in exploration of the enriched pathways in the context of their molecular interactions. This provides insight into the various signaling events triggered by the drugs that are discussed in the Results and Discussion sections.

#### Comparative pathway enrichment analysis using DAVID

For comparing our results with DAVID (https://david.ncifcrf.gov/) [5], we assigned median expression values of the biological replicates (samples) to the genes. Our default cutoff i.e. fold change of 2 (Log2 fold change = 1) was used to identify the differentially expressed genes.

We performed DAVID enrichment analysis using GO biological processes (GO_BP_FAT) and KEGG pathways as the annotation datasets. Significantly enriched processes based on similar criterion to that of eSkIN (i.e. *p*-value < 0.05), were considered for comparison.

## Results

To gain mechanistic insight into the action of BM and PC, skin-centric transcriptomic data analysis was performed using eSkIN (Refer Methods). It is well-known that BM is more effective in curbing inflammatory effects of AD but also known to cause more adverse effects especially on skin barrier formation as compared to PC [2]. Our analysis provides new insights in the form of detailed account of the pathways that explains the molecular perturbations after drug treatments. Tables 1 and 2 provide a brief account of our key findings that adds value to the previously reported findings by Jensen et al., 2012 [2]. The findings are further discussed elaborately in this section.

**Table 1:** Key findings from our analysis: Genes and pathways perturbed by BM

Gene/Pathway	Role in skin pathways	Impact derived from eSkIN
TNF pathway	TNF pathway via NF-κB regulates the transcription of inflammatory cytokines, adhesion molecules, MMP9 and SELE.	TNF and its receptors are downregulated after treatment with BM, thus effecting anti-inflammatory effect.
TLRs	TLRs play important role in inflammation by activating NF-κB, which in turn, activates inflammatory cytokines.	Downregulated after treatment with BM, thus bringing about anti-inflammatory effect.
IL4 pathway	Involved in T-cell and eosinophil chemotaxis	Downregulated after treatment with BM, thus contributes towards anti-inflammatory effect.
LOR, FLG, TGM5 and CDSN	Important skin barrier proteins	Upregulated after treatment with BM; contributes towards restoration of skin barrier functions.
IVL, Keratins, LCEs, desmocollins and desmogleins	Important skin barrier proteins	Downregulated after treatment with BM representing the damage to skin barrier; CD44, AKT1, PKC-δ, HRAS and MAP2K3 involved in pathway leading to transcriptional activation of barrier proteins are also downregulated in BM samples.
S100 family proteins	Important anti-microbial peptides that help in protecting the skin from infections.	Downregulated after treatment with BM, thus leading to impaired barrier function.
VEGF	Wound healing and cell migration, vascular permeability, angiogenesis, cell invasion and coagulation	Downregulated after treatment with BM, thus affecting wound healing and other cellular processes through PLC-γ and MAPK cascade.
H2AFX, RAD51, BRCA2, MCM3, DHFR, HMOX1, GINS1 and PCNA	Genes involved in DNA repair	Downregulated after treatment with BM, thus affecting DNA repair processes.

**Table 2:** Key findings from our analysis: Genes and pathways perturbed by PC

Gene/Pathway	Role in skin pathways	Impact derived from eSkIN
TGF-β	Plays important role in inflammation via SMADs, and regulates IFNG, IL2, CCL4, CXCL2 and MMP2 that are involved in T-cell chemotaxis and B-cell maturation	Downregulated after treatment with PC, thus contributes towards anti-inflammatory effect.
IL13 receptor (IL13RA2)	Important regulator of chemokines through JAK-STAT pathway	Downregulated after treatment with PC, thus contributes towards anti-inflammatory effect.
LOR, FLG, TGM5 and CDSN	Important skin barrier proteins	Upregulated after treatment with PC, thus contributes towards restoration of barrier functions.

### BM causes large-scale perturbations in inflammatory response as compared to PC

**Fig. 1:**
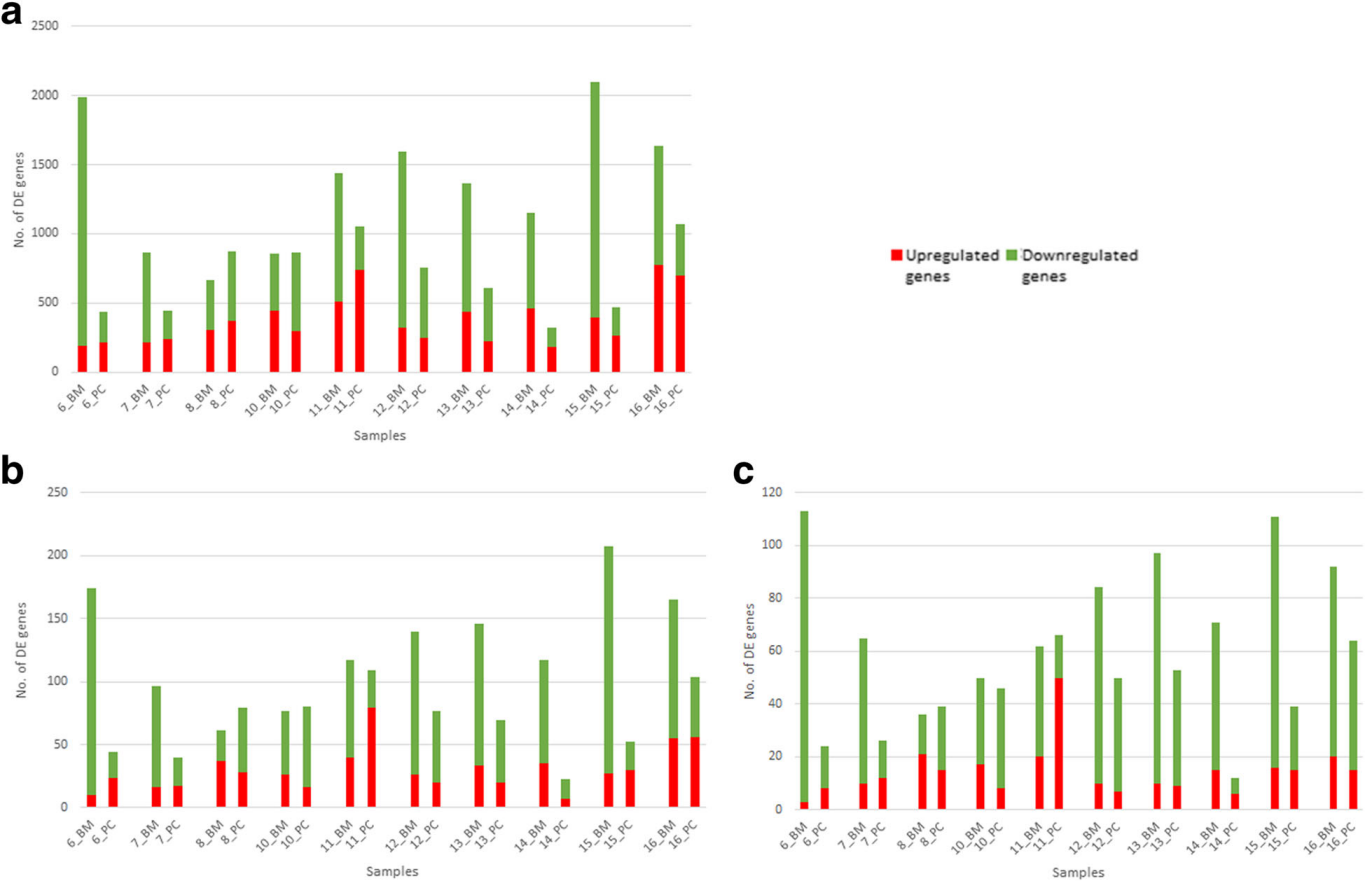
Differential gene expression in BM and PC samples. Differential gene expression with respect to (**a**) complete BM and PC datasets; (**b**) effect on Inflammation pathway; and (**c**) effect on Keratinocyte Differentiation pathway

As evident from Fig. 1a, the total number of differentially expressed genes (DEG) is higher in BM samples (approximately 1000–2000 genes) than that in PC samples (approximately 500–1000 genes), thus, indicating that BM has more profound effect on skin processes than PC. Similar trend is evident for Inflammation and Keratinocyte Differentiation pathways (see Fig. 1b-c).

**Fig. 2:**
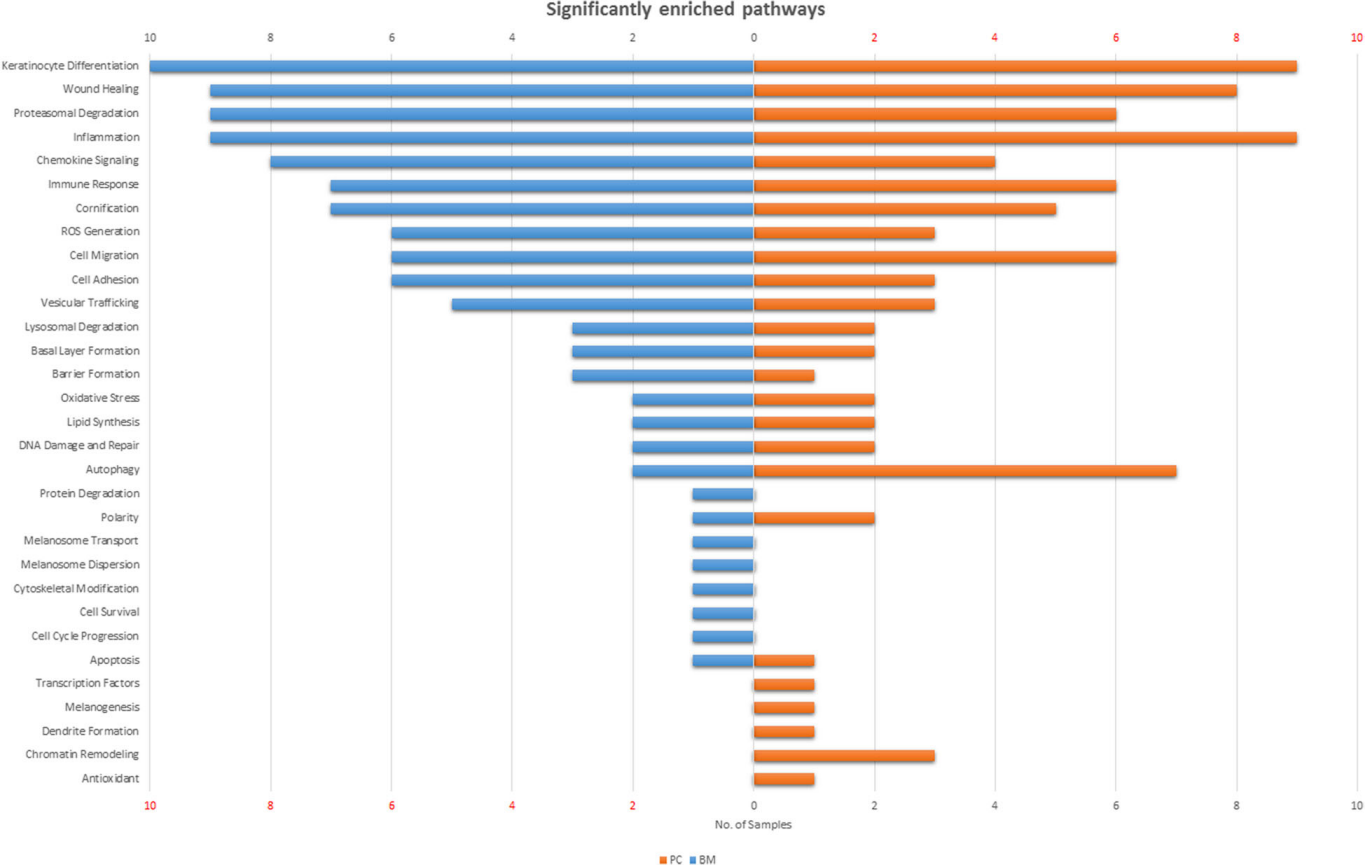
Significantly enriched eSkIN pathways in BM and PC samples. The length of the bars denote the number of samples in which the pathways are enriched. Pathways with eSkIN p-value < 0.05 are considered to be significantly enriched

Our pathway enrichment analysis shows that the pathways associated with inflammation (e.g. Inflammation, Immune Response and Chemokine Signaling) and skin barrier (e.g. Keratinocyte Differentiation, Wound Healing and Barrier Formation) are enriched (see Fig. 2), thus, corroborating the already reported findings. It is interesting to note that same set of pathways are enriched by both BM and PC, however, the detailed analysis clearly differentiates the mechanisms with which these drugs act on skin.

Additionally, pathways such as Basal Layer Formation and cellular pathways like Cell Migration, Cell Adhesion, Proteasomal Degradation, Autophagy, DNA Damage and Repair, Lipid Synthesis and ROS (Reactive Oxygen Species) Generation were also enriched in certain BM and PC samples.

#### BM mediates its anti-inflammatory effect via TNF, TLRs and IL4 pathways

BM, a corticosteroid, is known to activate glucocorticoid receptors (NR3C1) on skin, which in turn, inhibit the activity or transcription of various triggers of inflammation like IL1-β, IL4, IL11, TNF-α, TGF-β, MMPs (MMP1, 2 and 9), IFN-γ and VEGF [3]. Below, we present the inflammation-specific biomolecular interactions of these triggers in the context of transcriptomic data of BM treated samples, and relate them to key components of inflammatory response including the activation of T-cell, B-cell, eosinophils and monocytes.

**Fig. 3:**
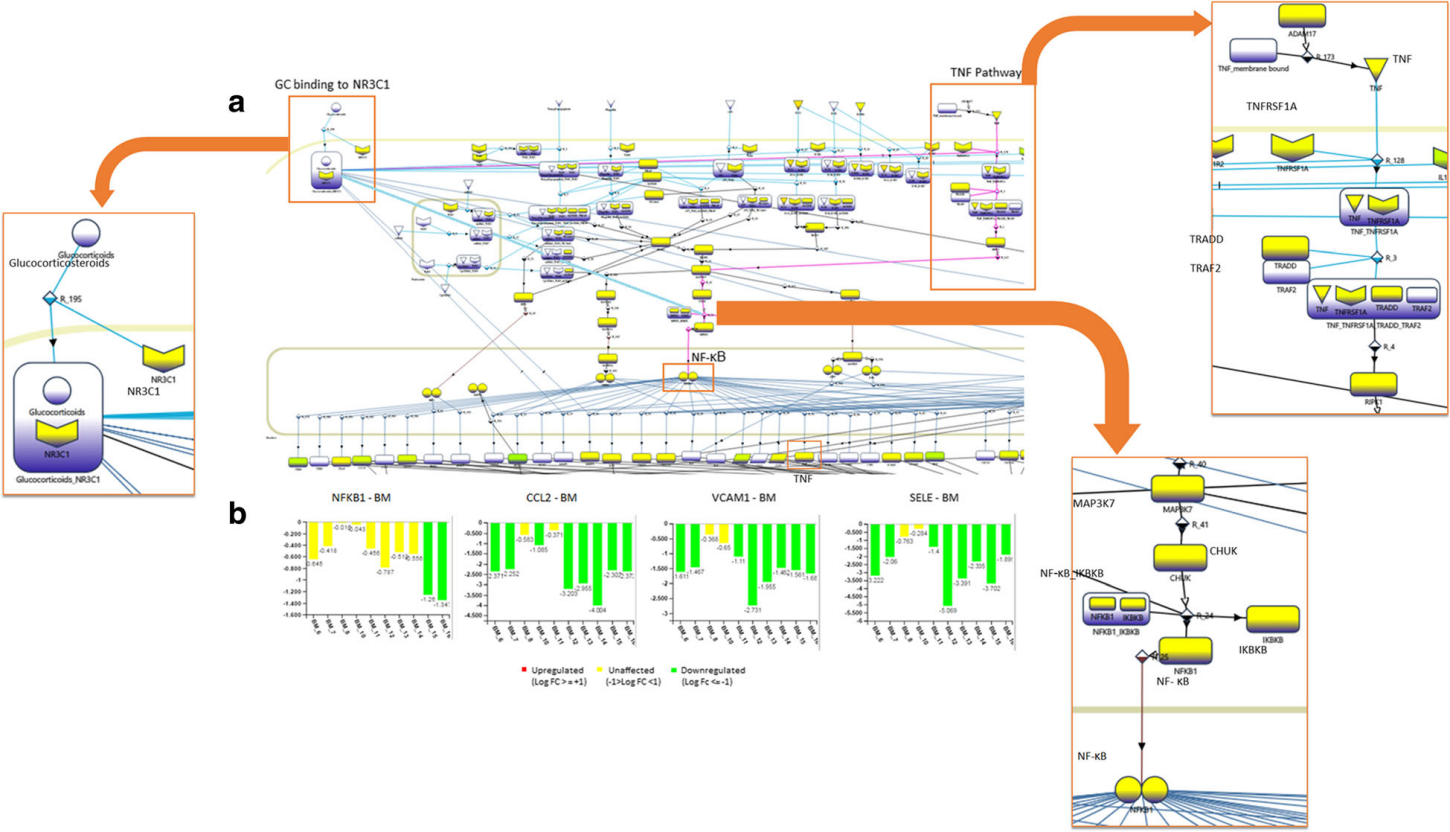
TNF mediated anti-inflammatory effect of BM. (**a**) Section of eSkIN Inflammation pathway showing NR3C1 mediated inhibition of TNF and the downstream repression of inflammatory markers via NF-κB; (**b**) Expression profiles showing various genes regulated by NF-κB. Important genes/proteins in the pathway are labelled for clarity

Our analysis of Inflammation pathway shows that NR3C1 can inhibit TNF pathway, albeit with an unclear mechanism [11]. Its involvement in anti-inflammatory response of BM is evident from the fact that TNF, its receptor TNFRSF1A and TRADD are downregulated (Additional file 5: Figure S2). TNF pathway is known to play a major role in activating NF-κB (NFKB1) [11], and thus, its inhibition results in deactivation of NF-κB (Fig. 3a).

NF-κB is one of the important factors in the transcription of inflammatory cytokines, and its deactivation affects inflammation by downregulating the expression of following genes: (i) inflammatory markers: CXCL1, CXCL9, CXCL10, IL18, CCL2, CCL5, CCL13 and CD86; (ii) adhesion molecules: ICAM1 and VCAM1; (iii) matrix metalloproteinases responsible for degradation of collagen: MMP9; and (iv) factors causing accumulation of leukocytes at the site of inflammation: SELE [12, 13]. Although NF-κB is significantly down regulated only in 2 out of 10 samples, it is indeed down regulated in all the other samples albeit in lower magnitude (see Fig. 3b and Additional file 6: Figure S3). However, as reported by Chen et al., a small change in expression level of NF-κB can result in significant change in the expression of its target genes [14], which is also observed in our study. Thus, this implicates NF-κB dependent anti-inflammatory effect as an important mechanism by which BM brings about its anti-inflammatory effect. Moreover, it is interesting to note that NF-κB also regulates the expression of TNF with the help of SMAD4 [12], thereby adding further to its anti-inflammatory effect.

eSkIN also shows that TLRs (TLR1 and TLR2), which are known to promote atopic dermatitis [15], are downregulated by BM. TLRs activate NF-κB through IRAK4, and thus downregulation of TLRs lead to suppression of inflammation by diminishing NF-κB dependent inflammation, as discussed above (Additional file 7: Figure S4) [16]. Moreover, eSkIN depicts that NR3C1 can also directly inhibit the activity of NF-κB through activation of IκB (IKBKB) [3]. However, IκB did not show significant change in its expression level in BM samples (Additional file 8: Figure S5), thus indicating that this is not a likely route taken by BM to bring about its anti-inflammatory effect.

eSkIN also depicts a route for NR3C1 mediated inhibition of IL4 pathway and IFN-γ (IFNG) pathway. Although, the transcriptomic data indicated downregulation of IL4 and its receptors in BM samples, IFN-γ pathway seems to be unaffected (Additional file 9: Figure S6a). Thus, implicating IL4 pathway as an effector of BM drug action. Our analysis indicate that IL4 pathway through JAK-STAT mechanism can regulate the transcription of CXCL6, CXCL16, CCL8, CCL24, CCL25 and CCL26 which play a major role in T-cell and eosinophil chemotaxis (Additional file 9: Figure S6b) [17]. The chemokines and cytokines downstream of this pathway are down regulated in most of the BM treated samples (Additional file 10: Figure S7). This indicates that IL4 mediated suppression of inflammation may also be contributing to anti-inflammatory effect of BM.

#### PC suppresses inflammation via IL13, IL6 and VEGF pathways

PC is a calcineurin inhibitor and inhibits the activity of NFAT (NFATC1) [4]. NFAT along with AP1 family of transcription factors is known to regulate the transcription of IL2, IL4, IL5, IL8, IL13, GM-CSF, TNF-α and IFN-γ, which are important inflammatory triggers [18, 19]. The transcriptomic data of PC samples show downregulation of IL13 receptor (IL13RA2), but, it also show downregulation of receptors of additional inflammatory triggers, namely IL6 and TGF-β. At the downstream, IL13 regulate the transcription of CCL8 and CCL26 via STAT6, and TGF-β regulate the transcription of CXCL2 via SMAD4. We observed all these downstream chemokines to be downregulated in PC samples (see Additional file 11: Figure S8). Moreover, the transcription of following chemokines and adhesion molecules: CXCL1, CXCL9, CXCL10, CCL2, SELE, ICAM1 and VCAM1, are also downregulated, but, their causal route could not be deciphered from this data.

### Additional pathways responsible for anti-inflammatory effect of BM and PC

- It is known that JAK-STAT cascade helps in the transcription of genes responsible for leukocyte, eosinophil and T-cell migration [20, 21]. Our analysis shows that BM and PC affects this activity through certain common triggers (i.e. IL6, IL4, and IL27) as is evident from downregulation of their respective receptors (in particular, IL6R, IL4R and IL27RA). PC also shows few specific triggers like IL13 and IL12 (downregulation of IL13RA2 and IL12RB) (Additional file 12: Figure S9).
- Chemokine Signaling is known to affect processes like adhesion, migration, phagocytosis, immune response and anaphylaxis. Our analysis shows that most of the signaling cascades like RAS-RAF pathway, MAPK cascade and JAK-STAT pathway, triggered by various chemokines and growth factors like HBEGF and AREG [22, 23], show profound under expression in BM samples but mild under expression in PC samples (data not shown).

### Restoration of skin barrier by BM and PC

Keratinocyte differentiation is central to the formation of healthy skin barrier. BM is known to efficiently inhibit the primary cause of AD i.e. inflammation, but it also affects the skin barrier leading to secondary complications like skin atrophy and infection.

Our analysis corroborates the fact that BM and PC upregulate several key skin barrier formation genes such as Loricrin (LOR), Filaggrin (FLG), TGM5 and CDSN (Additional file 13: Figure S10a) as a measure to restore barrier functions [2]. However, it is also observed that most of the other barrier formation proteins like involucrin (IVL), LCEs (LCE3D), TGM1, TGM3 and DSG3 are under expressed in BM samples while they are mostly unaffected by PC (Additional file 13: Figure S10b).

### BM adversely affects the synthesis of barrier proteins through AP1 family and CEBPs

**Fig. 4:**
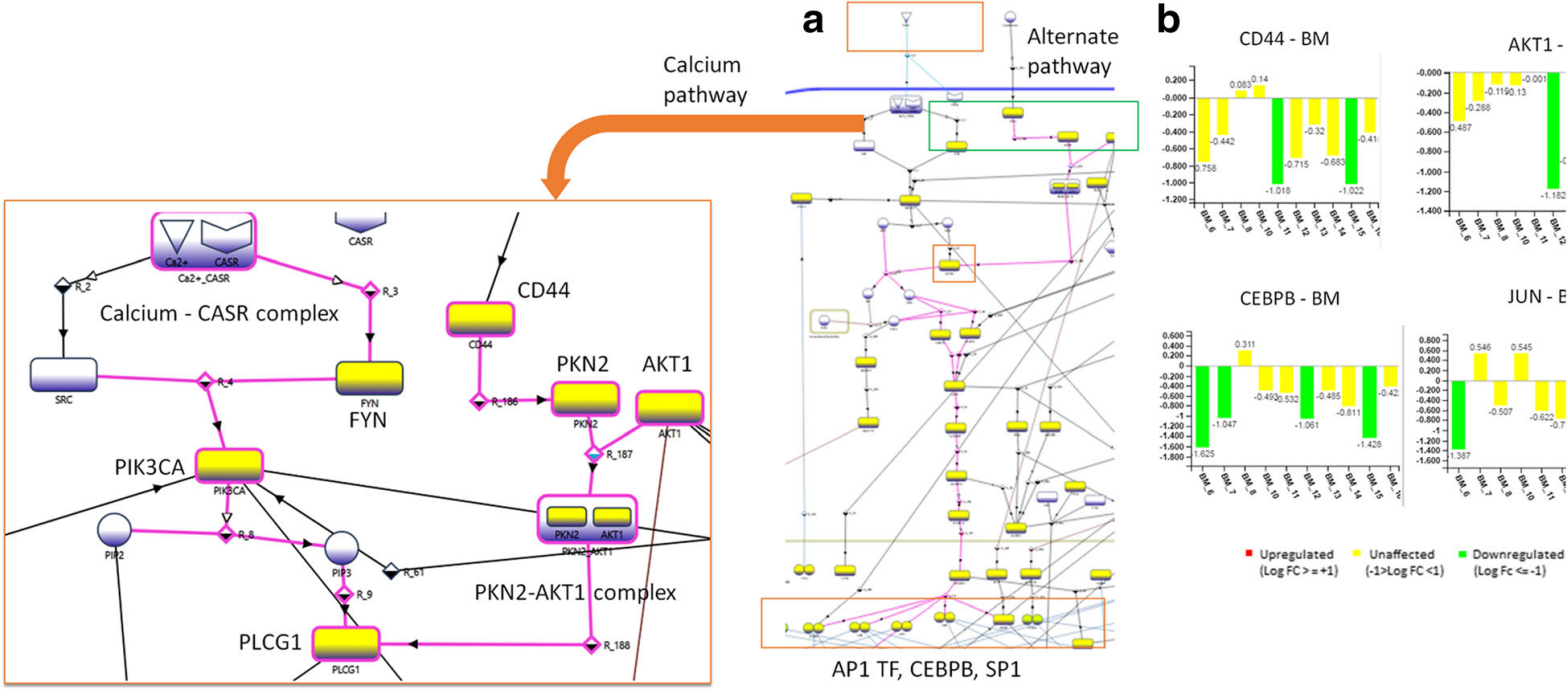
AP1 and CEBP mediated skin barrier impairment by BM. (**a**) eSkIN Keratinocyte Differentiation pathway depicting the effect of BM on synthesis of skin barrier proteins; (**b**) Expression profiles of key genes in this pathway. Important genes/proteins in the pathway are labelled for clarity

We explored the possible causative pathway that could lead to the impairment of skin barrier by BM. It is known that calcium acts as a key trigger for epidermal differentiation through PLC-γ (PLCG1), which regulates the transcription of barrier proteins. Our analysis depicts another, calcium independent route, to activate PLC-γ through AKT1-PKN2 complex which is activated by CD44 [24] (Fig. 4a). This pathway further activates several transcriptional regulators including AP1 family of transcription factors like JUN and FOS, CEBP-β (CEBPB), SP1 and HSPB1. These regulators play a major role in the transcription of barrier proteins like keratins, transglutaminases, loricrin, involucrin, filaggrin, LCEs, desmogleins, desmocollins, family of S100 proteins [25]. The molecular details feature of eSkIN shows that the genes involved in this pathway like CD44, AKT1, PKC-δ, HRAS and MAP2K3 are mostly downregulated in BM samples, thus, implying the role of this pathway in BM triggered skin barrier impairment (see Fig. 4b).

Other transcriptional regulators involved in the activation of skin barrier proteins like JUND and FOSL1, are also found to be downregulated. Furthermore, CEBP-α (CEBPA), an important transcription factor involved in the transcription of IVL and desmocollins [26], is also under expressed in BM samples (Additional file 14: Figure S11). TGFA pathway mediates this transcriptional regulation of CEBP-α [27]. The proliferation markers like MYC and ERK (MAPK1) present in this pathway are also under expressed in BM samples (data not shown).

Keratinocyte Differentiation pathway also indicates that the anti-microbial peptides S100A7, S100A8 and S100A9 are under expressed in BM samples (Additional file 15: Figure S12). While S100A7 transcription is mediated by JUN [28], S100A8 and S100A9 transcription is mediated by STAT3 via IL4 pathway (Additional file 15: Figure S12) [29]. This further explains the compromised skin barrier functions upon treatment with BM, leading to skin infection [2].

On the contrary, PC has mild effect on barrier proteins and their regulation, and thus, helps in restoring skin barrier in AD patients.

In addition to the profile of skin barrier proteins observed in Keratinocyte Differentiation pathway, Barrier Formation genes like CTTN, CDC42, GRHL3 and AHR which are involved in the formation of tight junctions [30] are under expressed in BM samples. PC samples does not show significant enrichment of this pathway in most of the samples (Additional file 16: Figure S13).

### Additional pathways responsible for effect on skin barrier by BM and PC

- Wound Healing pathway of eSkIN shows that VEGF (VEGFA), an important trigger for cellular processes like cell migration, vascular permeability, angiogenesis, cell invasion and coagulation, is significantly more under expressed in BM than in PC (Additional file 17: Figure S14). VEGFA activates various pathways like PI3K (PIK3CA), PLC-γ and MAPK cascade, thus, impacting the normal physiological processes that are responsible for wound healing [31]. Fibronectin (FN1), another protein involved in wound healing, is downregulated as well in BM samples [32].
- Similarly, Basal Layer Formation pathway shows that proteins like integrins, LAMA5 and collagens like COL4A1 [33] are under expressed in BM samples. On the contrary, most of these proteins show an upregulated trend in PC dataset (Additional file 18: Figure S15).

This further illustrates that BM affects the skin barrier formation by influencing the synthesis of barrier proteins, antimicrobial proteins and basal layer proteins. Such behavior is not observed in PC samples.

### Cellular functions affected by BM and PC

Lipid Synthesis pathway is of interest as lipids are an integral part of skin barrier. Application of BM is known to impair the fatty acids and lipid content of skin [34]. Lipid Synthesis pathway shows that lipid transporters like LDLR and ABCA12 [35, 36] show downregulated trend (Additional file 19: Figure S16a), which might hinder the transportation of cholesterol and other lipids to skin. Enzymes like SPTLC2, SGMS2 and SMPD2 involved in the conversion of fatty acids to glucosylceramides [36] are also downregulated (Additional file 19: Figure S16b). Moreover, genes involved in the synthesis of fatty acids or lipids like LXR (NR1H2), SCD, FASN and HMGCR [37, 38] are downregulated (Additional file 20: Figure S17a), while genes involved in the metabolism of lipids like LPL and APOC1 [39] are showing an upregulated trend (Additional file 20: Figure S17b).

DNA Damage and Repair is differentially affected in BM and PC dataset. While most of the genes are unaffected by PC, genes involved in DNA repair like H2AFX, RAD51, BRCA2, MCM3, DHFR, HMOX1, GINS1 and PCNA [40–42] are under expressed in BM (Additional file 21: Figure S18). Similarly, CDK1, CDKN1A, CCNB1 and E2F family of proteins, involved in cell cycle progression [43] show a downregulated trend in BM samples.

Though Autophagy and Proteasomal Degradation pathways are enriched in most of the BM and PC samples, we could not establish any relevance of this to the action of these drugs.

### Comparison of eSkIN results with DAVID

To evaluate the performance of eSkIN pathway enrichment analysis, we compared the results with the most widely used functional enrichment tool, DAVID (https://david.ncifcrf.gov/) [5]. BM and PC samples were analyzed separately in DAVID. We obtained 409 differentially expressed genes in BM and 49 genes in PC datasets that were used as input for DAVID analysis (refer Methods for details).

#### Comparison of pathway enrichment for BM dataset

The enriched processes for BM dataset yielded 781 GO Biological Processes (GO BP) terms and 24 KEGG pathways (see Additional file 22). We observe that Immune response, Defense response, Inflammatory response, Keratinocyte differentiation, Skin development and Keratinization are enriched amongst the GO terms. Amongst KEGG pathways, Drug metabolism, Steroid hormone biosynthesis, Chemokine signaling pathway and NF-kappa B signaling pathways are enriched.

eSkIN uses a comprehensive manually curated skin centric knowledge-base for its analysis, and thus we observe a limited set of only relevant pathways (26 pathways) to be enriched. The pathways related to skin physiology, which are enriched in DAVID analysis are also obtained using eSkIN analysis (see Table 3), thus, corroborating the capability of eSkIN to perform skin-centric pathway enrichment analysis.

**Table 3:** Comparison of pathways enriched in eSkIN and DAVID analysis

S.No.	Important pathways enriched in eSkIN	Related processes/pathways enriched in BM samples		Related processes/pathways enriched in PC samples	
		GO_BP_FAT	KEGG	GO_BP_FAT	KEGG
1.	Keratinocyte differentiation, Cornification, Basal layer formation	keratinocyte differentiation, skin development, keratinization, skin epidermis development	–	epidermis development, skin development, keratinocyte differentiation, epidermal cell differentiation, keratinization	–
2.	Inflammation	inflammatory response, regulation of inflammatory response, T-cell activation	TNF signaling pathway, NF-κB signaling pathway, Inflammatory bowel disease	–	–
3.	Immune response	Immune response, defense response, Innate immune response	–	Immune response, defense response, Innate immune response	–
4.	Chemokine signaling	Chemokine mediated signaling pathway and chemokine production	Chemokine signaling pathway, Cytokine-cytokine receptor interaction	–	–
5.	DNA Damage and Repair	DNA integrity checkpoint	–	–	–
6.	Cell Adhesion, Cell Migration	Cell adhesion, leukocyte cell-cell adhesion, cell migration, regulation of cell migration, leukocyte migration	Cell adhesion molecules (CAMs)	Cell migration, leukocyte migration	–

However, GO BP terms cannot be further explored in terms of the molecular interactions between these genes (or their protein products) to derive mechanistic insights into the action of the drugs. On the other hand, eSkIN allows further exploration of the enriched pathways in terms of the molecular interactions in them, and thus, helps in deriving mechanistic insights into drug action (see Table 1). Though KEGG Pathways can be explored in similar context, the enriched KEGG pathways (Drug metabolism, Steroid hormone biosynthesis Chemokine signaling pathway and NF-kappa B signaling pathways) are not directly relevant to our analysis.

#### Comparison of pathway enrichment for PC

The enriched processes for PC dataset yielded 62 GO BP terms and 0 KEGG pathways (see Additional file 22). We observe that skin related processes like Epidermis development, Skin development, Keratinocyte differentiation, Immune response and Defense response are enriched amongst the GO terms. As is evident from Table 3, the corresponding pathways are also enriched in eSkIN. Moreover, owing to the capability of eSkIN to highlight molecular interactions of the enriched pathways, insights into mechanistic action of PC were obtained (see Table 2).

## Discussion

**Fig. 5:**
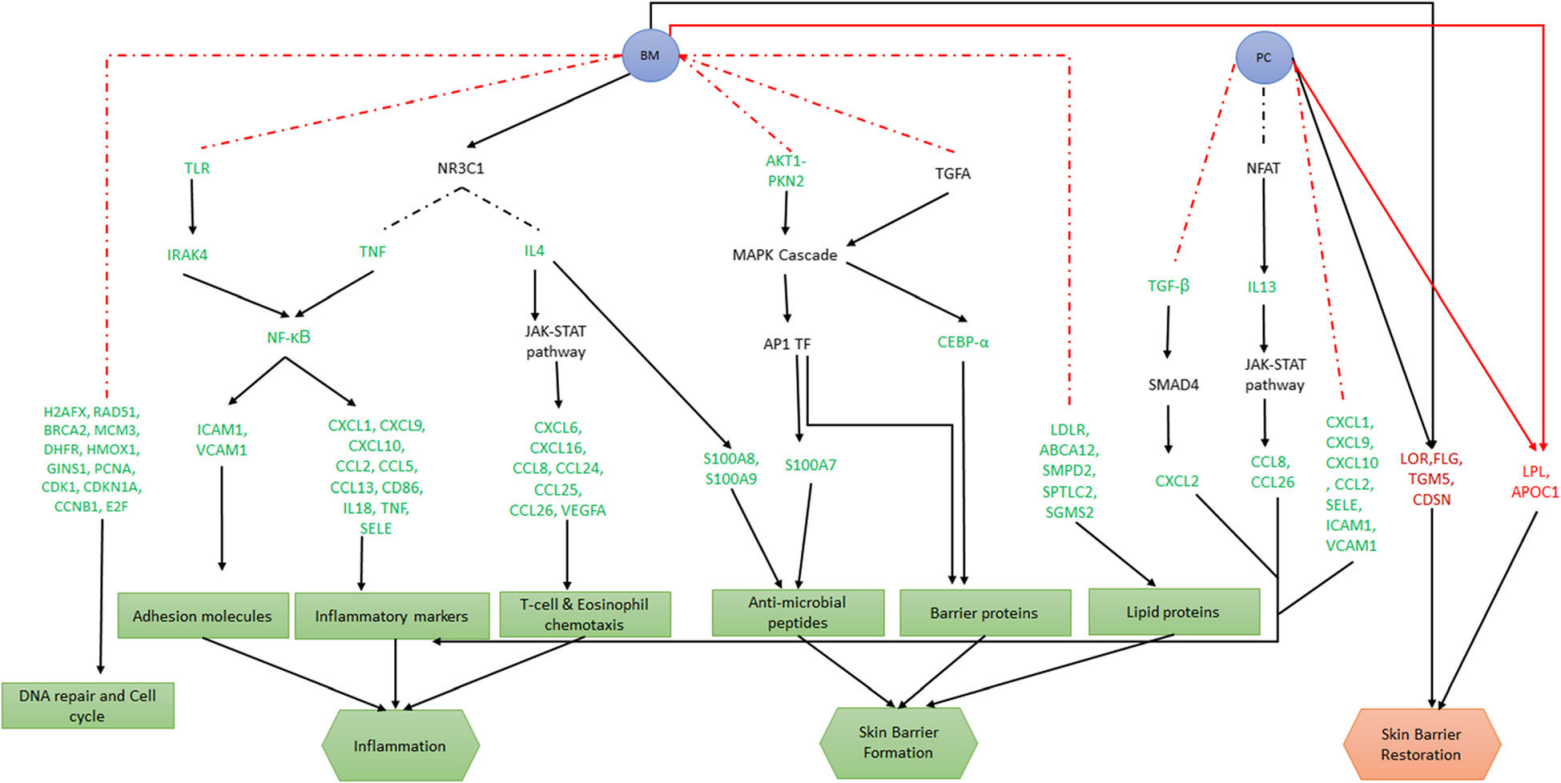
Pathways perturbed by BM and PC and their role in the action of these drugs. Solid lines depict activation or stimulation while the dotted lines represent inhibition. Colors of the lines denote known mechanism (black) and unknown mechanism (red). Colors of the gene names denote downregulation (green) and upregulation (red). This clearly shows that BM has a larger impact on inflammation than PC. But it is also evident that, unlike PC, BM negatively affects the skin barrier functions

In this study, we analyzed transcriptomic datasets of AD patients treated with Betamethasone and Pimecrolimus. We used a novel systems biology based approach to understand the detailed molecular level differences in the mechanism of action of the drugs. Though the pathway enrichment analysis broadly showed similar set of pathways being enriched by both BM and PC, a detailed molecular level study showed that these drugs opt different mechanisms to control the disorder. Fig. 5 provides an overview of the routes taken by these drugs.

We highlighted the role of TLRs, TNF and IL4 to trigger the anti-inflammatory response and AKT1-PKN2 and TGFA via MAPK cascade to affect the skin barrier proteins in AD patients, when treated with BM. Stojadinovic et al. have shown the role of glucocorticoids in various cellular processes like inflammation, innate immunity, cell migration, tissue remodeling, cell differentiation and cell death in keratinocytes [3]. Our analysis further adds value to the above mentioned finding by elaborating on the pathways that trigger various cellular responses, particularly in AD patients. We also depict the impact of BM on lipids and DNA damage and repair proteins thus leading to an impaired barrier function.

Under PC treatment, we show that TGF- and IL13 could be the plausible pathways involved in anti-inflammatory response. Through our comparative study of the two drugs, we show that the molecular signatures of barrier proteins under PC clearly contributes towards restoration of barrier.

### Novel insight into disease manifestation during drug treatment

The mechanistic analysis of transcriptomic data of BM and PC treated samples also allowed us to understand the manifestation of disease during the treatment by these drugs. This provides new avenue towards future direction of drug discovery efforts in this area.

**Fig. 6:**
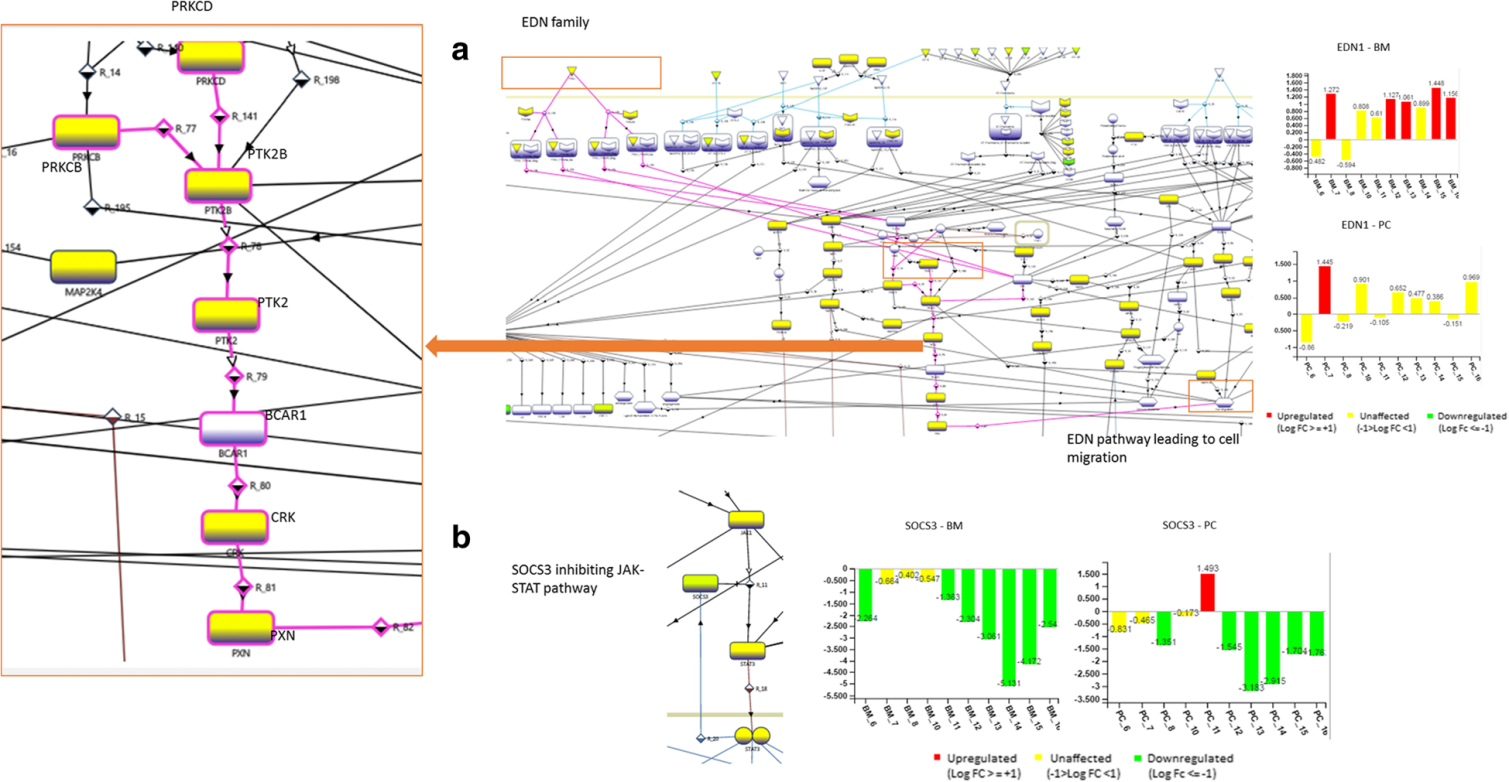
Factors implicated in manifestation of disease during drug treatment. (**a**) EDN1 mediated; (**b**) SOCS3 mediated. Important genes/proteins in the pathway are labelled for clarity

In particular, EDN1 (Endothelin 1), which is positively correlated with AD clinical severity [44, 45], is observed to be mostly upregulated in BM while PC shows mild upregulation. This implies a risk of disease manifestation even after treatment by BM or PC. By leveraging the molecular interaction maps of eSkIN, we elucidate that in the downstream, EDN1 activates PLCB2 [46], which in turn, triggers the MAPK cascade involved in cell migration and inflammation (see Fig. 6a). We believe that treatment by BM or PC, if supplemented with a drug targeting EDN1 or downstream proteins, can bring synergistic therapeutic effect in AD care.

Furthermore, it is interesting to note that the suppressors of cytokine family (SOCS), SOCS1 and SOCS3 are downregulated even after the treatment with BM and PC. SOCS proteins are known to contribute towards disease manifestation in psoriatic skin [47]. Our analysis shows that SOCS3 inhibits STAT3 activation via JAK1 [48] (Fig. 6b). However, their role in AD manifestation is not very well studied. Based on our analysis using eSkIN, it appears that downregulation of SOCS could be contributing towards the manifestation of AD, and thus, it may be worthwhile to explore it as a drug target for future drug discovery efforts.

## Conclusion

Our study suggested the causal biomolecular inter-relationships involved in the action of BM and PC on human skin, apart from highlighting the existing evidence on these drugs in the context of skin-associated functional networks. It is evident that BM downregulates molecules involved in inflammation, T-cell & eosinophil chemotaxis, adhesion, DNA repair and cell cycle, while PC targets a smaller section of inflammatory genes. Also, both BM and PC upregulate few important barrier proteins to restore the skin barrier functions. However, BM also downregulates many other barrier components which is largely unaffected by PC, thus, accounting for the impaired skin barrier due to the treatment with BM. It should be noted that the results reported in this study are based on 10 AD patients’ data that were available in NCBI GEO database, and needs further validation in a larger cohort.

## Additional files

Additional file 1: **Table S1.** List of 35 pathways of eSkIN and their categorization. (DOCX 13 kb)

Additional file 2: Sensitivity analysis to evaluate the effect of fold change cutoff on pathway enrichment analysis. (DOCX 12 kb)

Additional file 3: Pathway enrichment analysis for different fold change cutoffs. Table and charts of pathway enrichment analysis for different fold change cutoffs. (XLSX 30 kb)

Additional file 4: **Figure S1.** Comparison of enriched pathways at three different fold change cutoffs (Log_2_ fold change (FC) = 0.5, 1 and 1.5). (TIFF 3928 kb)

Additional file 5: **Figure S2.** Expression profile of genes involved in TNF pathway in BM samples. (TIFF 4333 kb)

Additional file 6: **Figure S3.** Expression profile of genes regulated by NF-κB in BM samples. (TIFF 12640 kb)

Additional file 7: **Figure S4.** Section of eSkIN Inflammation pathway showing TLR mediated activation of NF-κB and the expression profile of genes involved in this pathway. (TIFF 12017 kb)

Additional file 8: **Figure S5.** Section of eSkIN Inflammation pathway showing NR3C1 mediated inhibition of NF-κB through IκB (highlighted in pink) and the expression profile of IκB. (TIFF 7299 kb)

Additional file 9: **Figure S6.** Sections of eSkIN Inflammation pathway showing: (a) activation of JAK-STAT pathway by IL4 and IFN-γ and their expression profiles (b) genes regulated by STAT6. (TIFF 9845 kb)

Additional file 10: **Figure S7.** Expression profile of genes activated by IL4 via JAK-STAT pathway. (TIFF 5004 kb)

Additional file 11: **Figure S8.** Expression profile of inflammatory genes that show downregulation in PC samples. (TIFF 10440 kb)

Additional file 12: **Figure S9.** Section of eSkIN Immune Response pathway showing various activators of JAK-STAT pathway and their expression profile in BM and PC samples. (TIFF 10437 kb)

Additional file 13: **Figure S10.** Expression profile of skin barrier proteins in BM and PC samples. (a) Expression profile of genes that are upregulated in both BM and PC in order to restore barrier functions; (b) Expression profile of skin barrier genes that show treatment specific difference in their expressions. (TIFF 8378 kb)

Additional file 14: **Figure S11.** Expression profile of important transcription factors of barrier proteins in BM. (TIFF 5538 kb)

Additional file 15: **Figure S12.** Expression profile of anti-microbial peptides in BM samples and a section of eSkIN pathway showing their transcriptional regulation by IL4. (TIFF 5747 kb)

Additional file 16: **Figure S13.** Expression profile of junction proteins that show treatment specific difference in their expressions. (TIFF 6474 kb)

Additional file 17: **Figure S14.** Section of eSkIN Wound Healing pathway showing VEGF mediated activation of cellular functions like focal adhesion, actin remodeling, cell migration, vascular permeability, angiogenesis, degradation of collagen, cell invasion and blood coagulation, and expression profile of VEGF in BM and PC samples. (TIFF 12210 kb)

Additional file 18: **Figure S15.** Expression profile of basal layer genes that show treatment specific difference in their expressions. (TIFF 12463 kb)

Additional file 19: **Figure S16.** Sections of eSkIN Lipid Synthesis pathway showing: (a) lipid transporters and their expression profiles in BM samples (b) enzymes involved in fatty acid conversion and their expression profiles in BM samples. (TIFF 5067 kb)

Additional file 20: **Figure S17.** Sections of eSkIN Lipid Synthesis pathway showing: (a) the genes involved in the synthesis of lipids and fatty acids and their expression profiles in BM samples (b) genes involved in lipid metabolism and their expression profiles in BM samples. (TIFF 7877 kb)

Additional file 21: **Figure S18.** Section of eSkIN DNA Damage and Repair pathway showing the genes involved in DNA repair mechanisms and their expression profiles in BM samples. (TIFF 10956 kb)

Additional file 22: Pathway enrichment analysis using DAVID. Tables of enriched pathways (*p*-value < 0.05) using GO biological process (GO_BP_FAT) and KEGG pathways as annotation datasets in DAVID. BM and PC datasets were analyzed separately. (XLSX 168 kb)

## Abbreviations

AD: Atopic dermatitis
BM: Betamethasone valerate
DEG: Differentially expressed genes
GEO: Gene Expression Omnibus
PC: Pimecrolimus
ROS: Reactive oxygen species
TCI: Topical calcineurin inhibitor
US FDA: US Food and Drug Administration

## References

[1] Nutten S (2015). Atopic dermatitis: global epidemiology and risk factors. Ann Nutr Metab.

[2] Jensen JM (2012). Gene expression is differently affected by pimecrolimus and betamethasone in lesional skin of atopic dermatitis. Allergy.

[3] Stojadinovic O (2007). Novel genomic effects of glucocorticoids in epidermal keratinocytes: inhibition of apoptosis, interferon-gamma pathway, and wound healing along with promotion of terminal differentiation. J Biol Chem.

[4] Siegfried EC, Jaworski JC, Hebert AA (2013). Topical calcineurin inhibitors and lymphoma risk: evidence update with implications for daily practice. Am J Clin Dermatol.

[5] Huang DW, Sherman BT, Lempicki RA (2009). Systematic and integrative analysis of large gene lists using DAVID bioinformatics resources. Nat Protoc.

[6] Huang DW, Sherman BT, Lempicki RA (2009). Bioinformatics enrichment tools: paths toward the comprehensive functional analysis of large gene lists. Nucleic Acids Res.

[7] Subramanian A (2005). Gene set enrichment analysis: a knowledge-based approach for interpreting genome-wide expression profiles. Proc Natl Acad Sci U S A.

[8] J.-H. Hung, “Gene set/pathway enrichment analysis,” in Data Mining for Systems Biology, H. Mamitsuka, C. DeLisi, and M. Kanehisa, Eds. Humana Press, 2013, pp. 201–213.

[9] P. Zhao *et al.*, “Identification of differentially expressed genes in pituitary adenomas by integrating analysis of microarray data,” Int J Endocrinol, 2015. Available: https://www.hindawi.com/journals/ije/2015/164087/. Accessed 17 Oct 2017. 10.1155/2015/164087 PMC4302352 25642247

[10] Wu X (2016). Network expansion and pathway enrichment analysis towards biologically significant findings from microarrays. J Integr Bioinforma.

[11] Bradley JR (2008). TNF-mediated inflammatory disease. J Pathol.

[12] Miller LS (2008). Toll-like receptors in skin. Adv Dermatol.

[13] Murphy JE, Robert C, Kupper TS (2000). Interleukin-1 and cutaneous inflammation: a crucial link between innate and acquired immunity. J. Invest. Dermatol..

[14] Chen Y (2010). Microarray analysis reveals the inhibition of nuclear factor-kappa B signaling by aristolochic acid in normal human kidney (HK-2) cells. Acta Pharmacol Sin.

[15] Kaesler S (2014). Toll-like receptor 2 ligands promote chronic atopic dermatitis through IL-4-mediated suppression of IL-10. J Allergy Clin Immunol.

[16] Hari A, Flach TL, Shi Y, Mydlarski PR (2010). Toll-like receptors: role in dermatological disease. Mediat Inflamm.

[17] Bao L, Zhang H, Chan LS (2013). The involvement of the JAK-STAT signaling pathway in chronic inflammatory skin disease atopic dermatitis. JAK-STAT.

[18] Im S-H, Rao A (2004). Activation and deactivation of gene expression by Ca2+/calcineurin-NFAT-mediated signaling. Mol Cells.

[19] Medyouf H, Ghysdael J (2008). The calcineurin/NFAT signaling pathway: a novel therapeutic target in leukemia and solid tumors. Cell Cycle Georget Tex.

[20] Murata T, Husain SR, Mohri H, Puri RK (1998). Two different IL-13 receptor chains are expressed in normal human skin fibroblasts, and IL-4 and IL-13 mediate signal transduction through a common pathway. Int Immunol.

[21] Wittmann M, Zeitvogel J, Wang D, Werfel T (2009). IL-27 is expressed in chronic human eczematous skin lesions and stimulates human keratinocytes. J Allergy Clin Immunol.

[22] New DC, Wong YH (2003). CC chemokine receptor-coupled signalling pathways. Sheng Wu Hua Xue Yu Sheng Wu Wu Li Xue Bao (Shanghai).

[23] Pastore S, Mascia F, Mariotti F, Dattilo C, Mariani V, Girolomoni G (2005). ERK1/2 regulates epidermal chemokine expression and skin inflammation. J Immunol Baltim Md 1950.

[24] Bourguignon LYW, Singleton PA, Diedrich F (2004). Hyaluronan-CD44 interaction with Rac1-dependent protein kinase N-gamma promotes phospholipase Cgamma1 activation, ca(2+) signaling, and cortactin-cytoskeleton function leading to keratinocyte adhesion and differentiation. J Biol Chem.

[25] Eckert RL (2004). Regulation of involucrin gene expression. J. Invest. Dermatol..

[26] Efimova T, Deucher A, Kuroki T, Ohba M, Eckert RL (2002). Novel protein kinase C isoforms regulate human keratinocyte differentiation by activating a p38 delta mitogen-activated protein kinase cascade that targets CCAAT/enhancer-binding protein alpha. J Biol Chem.

[27] Reynolds NJ (1993). Differential induction of phosphatidylcholine hydrolysis, diacylglycerol formation and protein kinase C activation by epidermal growth factor and transforming growth factor-alpha in normal human skin fibroblasts and keratinocytes. Biochem J.

[28] Emberley ED (2005). The S100A7-c-Jun activation domain binding protein 1 pathway enhances prosurvival pathways in breast cancer. Cancer Res.

[29] Uto-Konomi A (2012). Dysregulation of suppressor of cytokine signaling 3 in keratinocytes causes skin inflammation mediated by interleukin-20 receptor-related cytokines. PLoS One.

[30] Jamora C, Fuchs E (2002). Intercellular adhesion, signalling and the cytoskeleton. Nat Cell Biol.

[31] Olsson A-K, Dimberg A, Kreuger J, Claesson-Welsh L (2006). VEGF receptor signalling - in control of vascular function. Nat Rev Mol Cell Biol.

[32] Zambruno G (1995). Transforming growth factor-beta 1 modulates beta 1 and beta 5 integrin receptors and induces the de novo expression of the alpha v beta 6 heterodimer in normal human keratinocytes: implications for wound healing. J Cell Biol.

[33] Fleischmajer R (1998). Initiation of skin basement membrane formation at the epidermo-dermal interface involves assembly of laminins through binding to cell membrane receptors. J Cell Sci.

[34] Jensen J-M (2009). Different effects of pimecrolimus and betamethasone on the skin barrier in patients with atopic dermatitis. J Allergy Clin Immunol.

[35] Zhang L, Reue K, Fong LG, Young SG, Tontonoz P (2012). Feedback regulation of cholesterol uptake by the LXR-IDOL-LDLR axis. Arterioscler Thromb Vasc Biol.

[36] Oda Y, Uchida Y, Moradian S, Crumrine D, Elias PM, Bikle DD (2009). Vitamin D receptor and coactivators SRC2 and 3 regulate epidermis-specific sphingolipid production and permeability barrier formation. J. Invest. Dermatol..

[37] Yokoyama A (2009). Induction of SREBP-1c mRNA by differentiation and LXR ligand in human keratinocytes. J. Invest. Dermatol..

[38] Harris IR (1998). Parallel regulation of sterol regulatory element binding protein-2 and the enzymes of cholesterol and fatty acid synthesis but not ceramide synthesis in cultured human keratinocytes and murine epidermis. J Lipid Res.

[39] Jiang ZG, Robson SC, Yao Z (2013). Lipoprotein metabolism in nonalcoholic fatty liver disease. J Biomed Res.

[40] Malewicz M (2011). Essential role for DNA-PK-mediated phosphorylation of NR4A nuclear orphan receptors in DNA double-strand break repair. Genes Dev.

[41] Yoshida K, Miki Y (2004). Role of BRCA1 and BRCA2 as regulators of DNA repair, transcription, and cell cycle in response to DNA damage. Cancer Sci.

[42] Meng X, Yuan Y, Maestas A, Shen Z (2004). Recovery from DNA damage-induced G2 arrest requires actin-binding protein filamin-a/actin-binding protein 280. J Biol Chem.

[43] Sulaimon SS, Kitchell BE (2003). The basic biology of malignant melanoma: molecular mechanisms of disease progression and comparative aspects. J Vet Intern Med.

[44] Aktar MK, Kido-Nakahara M, Furue M, Nakahara T (2015). Mutual upregulation of endothelin-1 and IL-25 in atopic dermatitis. Allergy.

[45] Tsybikov NN, Petrisheva IV, Kuznik BI, Magen E (2015). Plasma endothelin-1 levels during exacerbation of atopic dermatitis. Allergy Asthma Proc.

[46] Bouallegue A, Daou GB, Srivastava AK (2007). Endothelin-1-induced signaling pathways in vascular smooth muscle cells. Curr Vasc Pharmacol.

[47] Sonkoly E (2007). MicroRNAs: novel regulators involved in the pathogenesis of psoriasis?. PLoS One.

[48] Yamasaki K (2003). Suppressor of cytokine signaling 1/JAB and suppressor of cytokine signaling 3/cytokine-inducible SH2 containing protein 3 negatively regulate the signal transducers and activators of transcription signaling pathway in normal human epidermal keratinocytes. J Invest Dermatol.

