# Supplementary material for: Elucidating mechanistic insights into drug action for atopic dermatitis: a systems biology approach
Source: BMC Dermatol. 2018 Feb 7;18:3. doi: 10.1186/s12895-018-0070-4 (PMC5803917; doi:10.1186/s12895-018-0070-4)
Supplement: Supplementary file 1 — Table S1. List of 35 pathways of eSkIN and their categorization. (DOCX 13 kb) [file 12895_2018_70_MOESM1_ESM.docx]

**Table S1**: List of 35 pathways of eSkIN and their categorization

| **Category** | **Pathway** |
| --- | --- |
| Epidermis Formation | Barrier Formation |
|  | Basal Layer Formation |
|  | Cornification |
|  | Keratinocyte Differentiation |
|  | Lipid Synthesis |
| Skin Physiology | Apoptosis |
|  | Autophagy |
|  | Cell Adhesion |
|  | Cell Cycle Progression |
|  | Cell Migration |
|  | Cell Survival |
|  | Chemokine Signaling |
|  | Chromatin Remodeling |
|  | Cytoskeletal Modification |
|  | Lysosomal Degradation |
|  | Membrane Reorganization |
|  | Polarity |
|  | Proteasomal Degradation |
|  | Protein Degradation |
|  | Protein Turnover |
|  | Transcription Factors |
|  | Water Retention |
| Skin Pigmentation | Dendrite Formation |
|  | Melanogenesis |
|  | Melanosome Biogenesis |
|  | Melanosome Dispersion |
|  | Melanosome Transport |
|  | Vesicular Trafficking |
| Stress Pathways | Antioxidant |
|  | DNA Damage and Repair |
|  | Immune Response |
|  | Inflammation |
|  | Oxidative Stress |
|  | ROS Generation |
|  | Wound Healing |
