# Supplementary material for: Elucidating mechanistic insights into drug action for atopic dermatitis: a systems biology approach
Source: BMC Dermatol. 2018 Feb 7;18:3. doi: 10.1186/s12895-018-0070-4 (PMC5803917; doi:10.1186/s12895-018-0070-4)
Supplement: Supplementary file 2 — Sensitivity analysis to evaluate the effect of fold change cutoff on pathway enrichment analysis. (DOCX 12 kb) [file 12895_2018_70_MOESM2_ESM.docx]

**Sensitivity analysis to evaluate the effect of fold change cutoff on pathway enrichment analysis**

To evaluate the effect of fold change cutoff on our pathway enrichment analysis, we performed the analysis by varying our default cutoff (i.e. Log_2_ fold change = 1). The cutoff was increased by one fold change (i.e. Log_2_ fold change = 1.5) and reduced by one fold change (i.e. Log_2_ fold change = 0.5) of the default value. We observe that important pathways associated with inflammation (e.g. Inflammation, Immune Response and Chemokine Signaling) and skin barrier (e.g. Keratinocyte Differentiation, Wound Healing and Barrier Formation) are enriched at all the three fold change cutoffs in more than 50% of the samples (see Additional File 3). Moreover, it is observed that 24 out of 35 eSkIN pathways are enriched in at least one sample at all the fold change cutoffs (see Additional File 3 and Additional File 4: Figure S1). The three pathways that were observed to be enriched at only one of the cutoffs, are enriched in less than 50% of the samples. Thus, the analysis shows that the key pathways contributing towards the drug action are captured by all the three fold change cutoffs.
