# Supplementary figures and images for: Elucidating mechanistic insights into drug action for atopic dermatitis: a systems biology approach
Source: BMC Dermatol. 2018 Feb 7;18:3. doi: 10.1186/s12895-018-0070-4 (PMC5803917; doi:10.1186/s12895-018-0070-4)

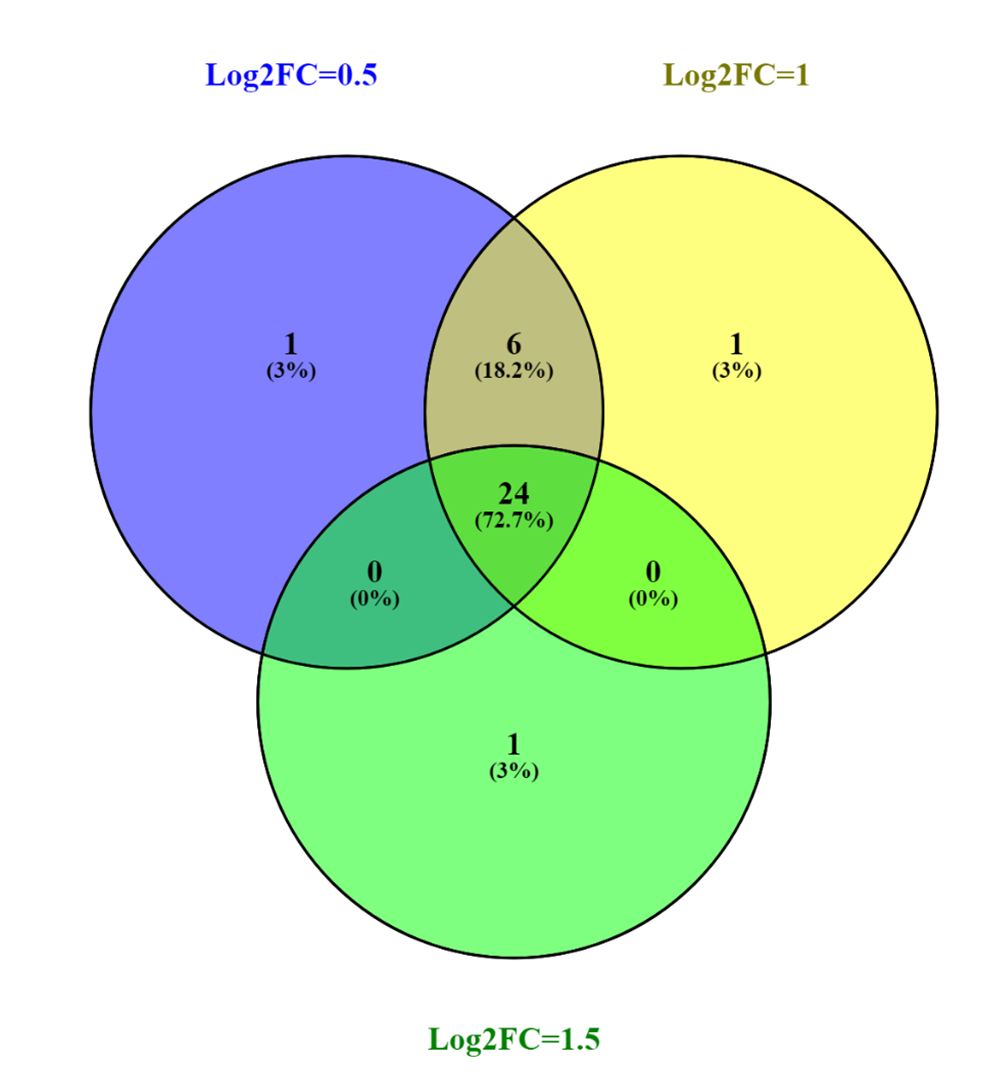

Supplement: Supplementary file 4 — Figure S1. Comparison of enriched pathways at three different fold change cutoffs (Log2 fold change (FC) = 0.5, 1 and 1.5). (TIFF 3928 kb) [file 12895_2018_70_MOESM4_ESM.tif]

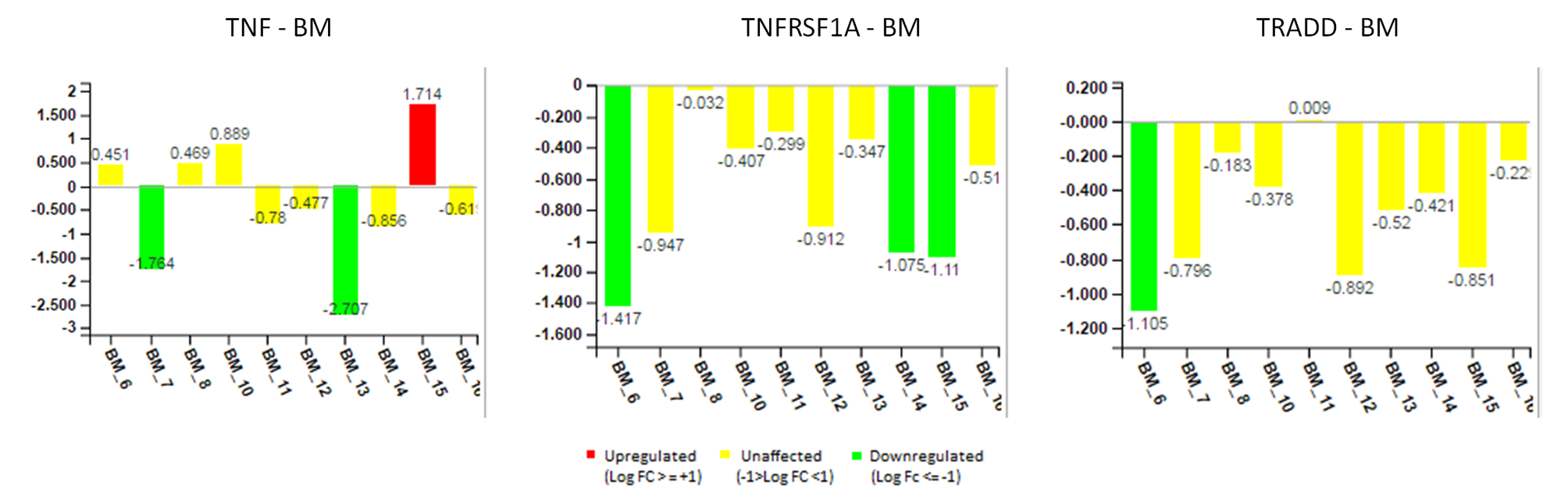

Supplement: Supplementary file 5 — Figure S2. Expression profile of genes involved in TNF pathway in BM samples. (TIFF 4333 kb) [file 12895_2018_70_MOESM5_ESM.tif]

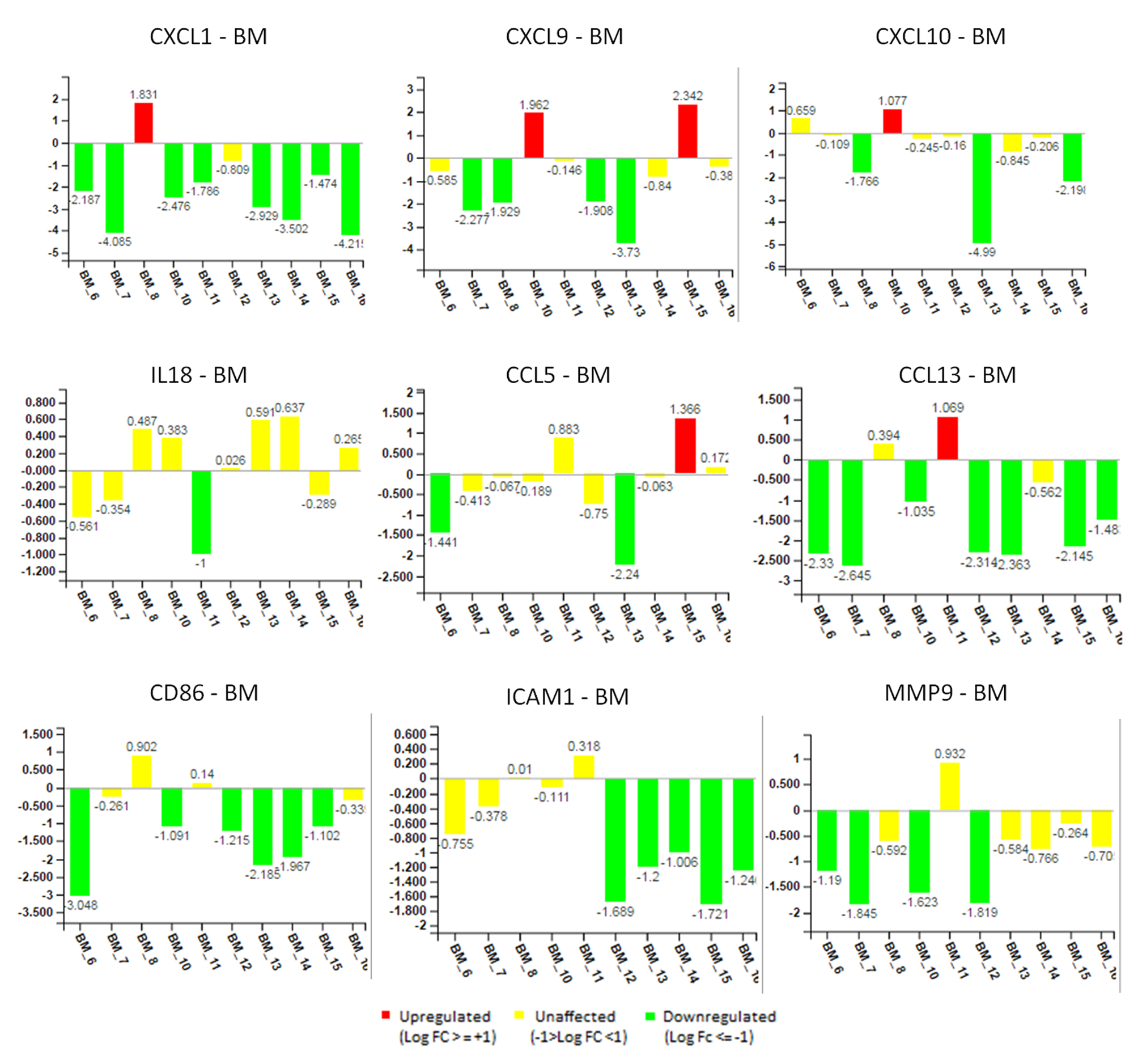

Supplement: Supplementary file 6 — Figure S3. Expression profile of genes regulated by NF-κB in BM samples. (TIFF 12640 kb) [file 12895_2018_70_MOESM6_ESM.tif]

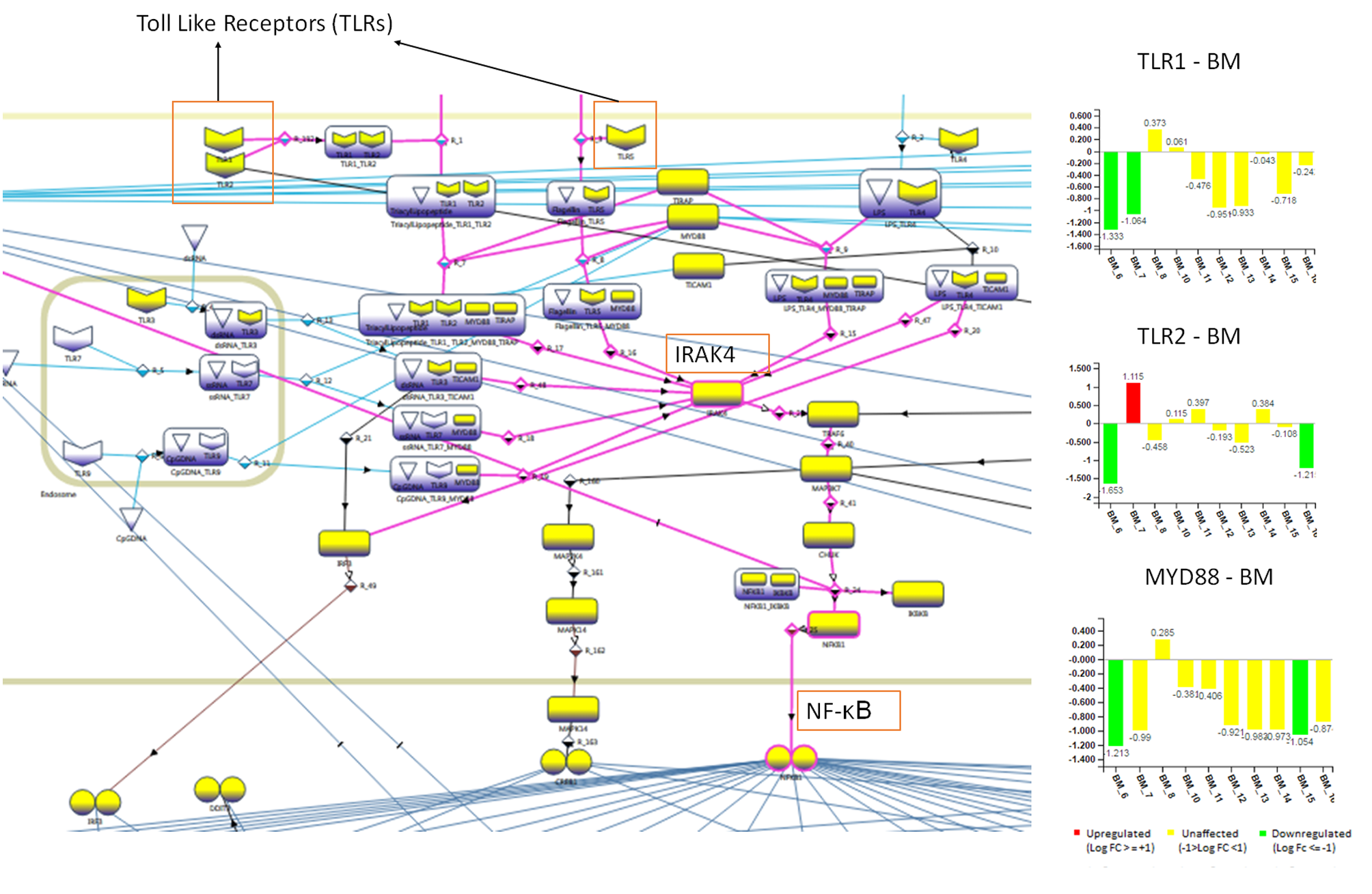

Supplement: Supplementary file 7 — Figure S4. Section of eSkIN Inflammation pathway showing TLR mediated activation of NF-κB and the expression profile of genes involved in this pathway. (TIFF 12017 kb) [file 12895_2018_70_MOESM7_ESM.tif]

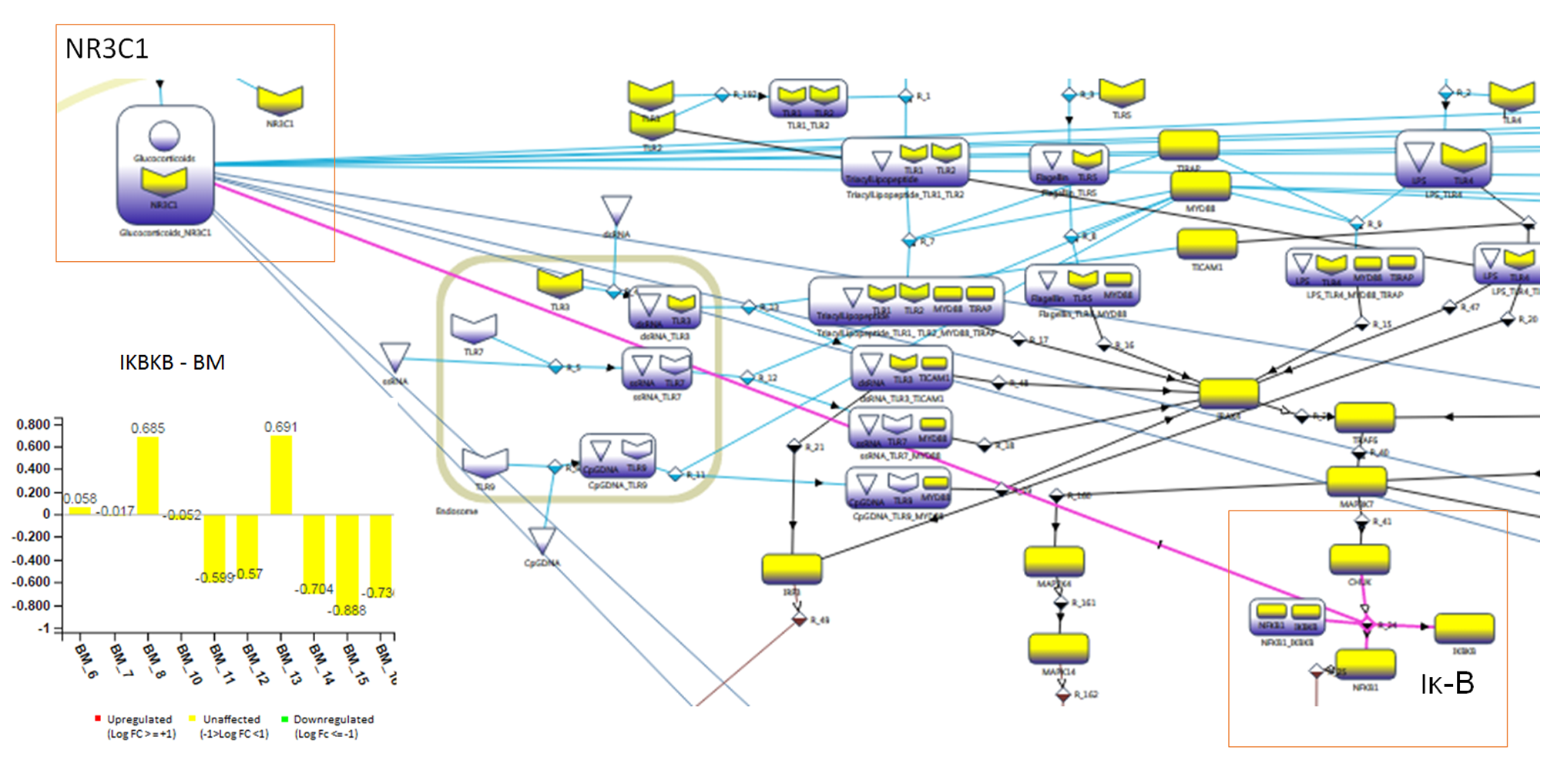

Supplement: Supplementary file 8 — Figure S5. Section of eSkIN Inflammation pathway showing NR3C1 mediated inhibition of NF-κB through IκB (highlighted in pink) and the expression profile of IκB. (TIFF 7299 kb) [file 12895_2018_70_MOESM8_ESM.tif]

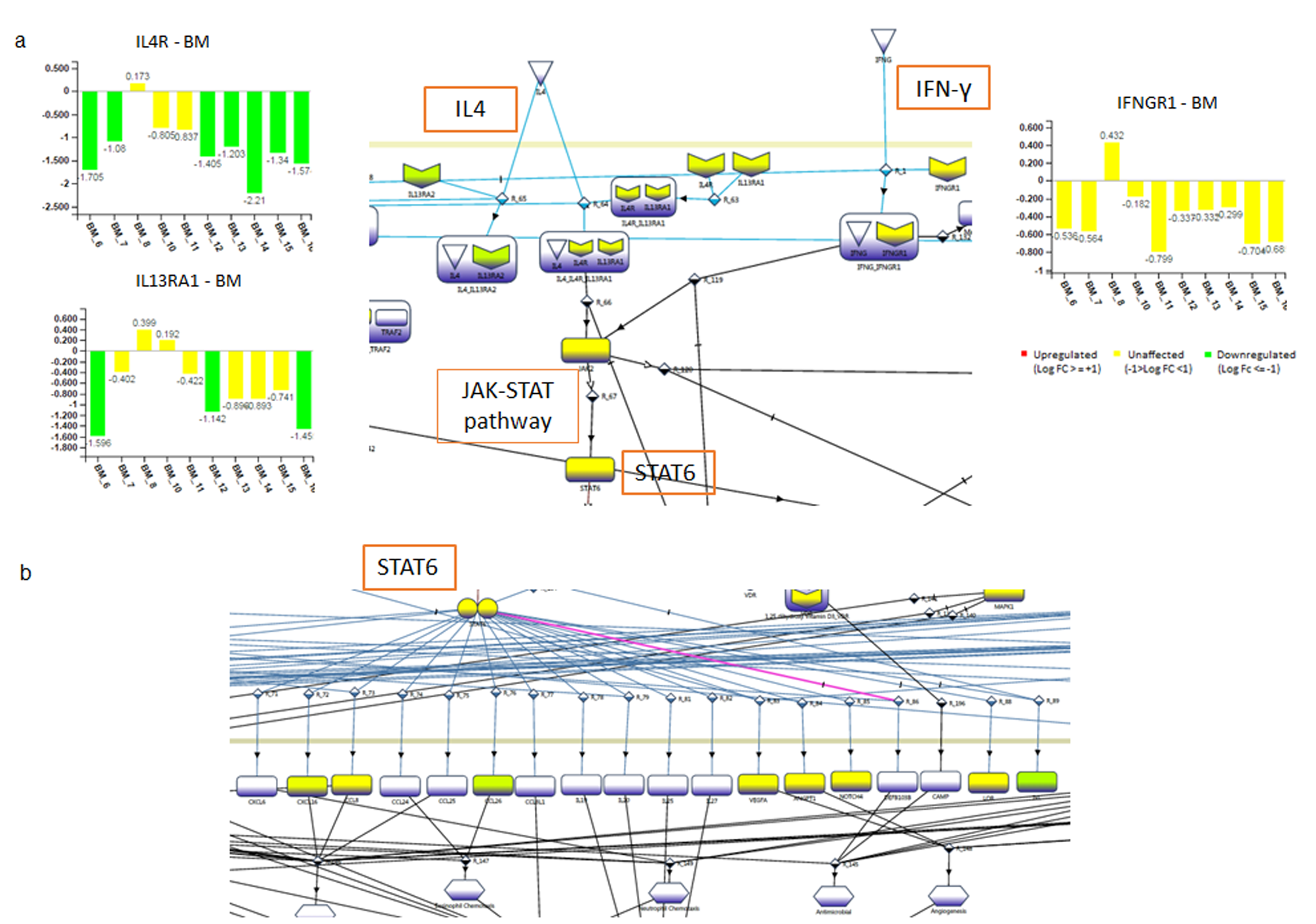

Supplement: Supplementary file 9 — Figure S6. Sections of eSkIN Inflammation pathway showing: (a) activation of JAK-STAT pathway by IL4 and IFN-γ and their expression profiles (b) genes regulated by STAT6. (TIFF 9845 kb) [file 12895_2018_70_MOESM9_ESM.tif]

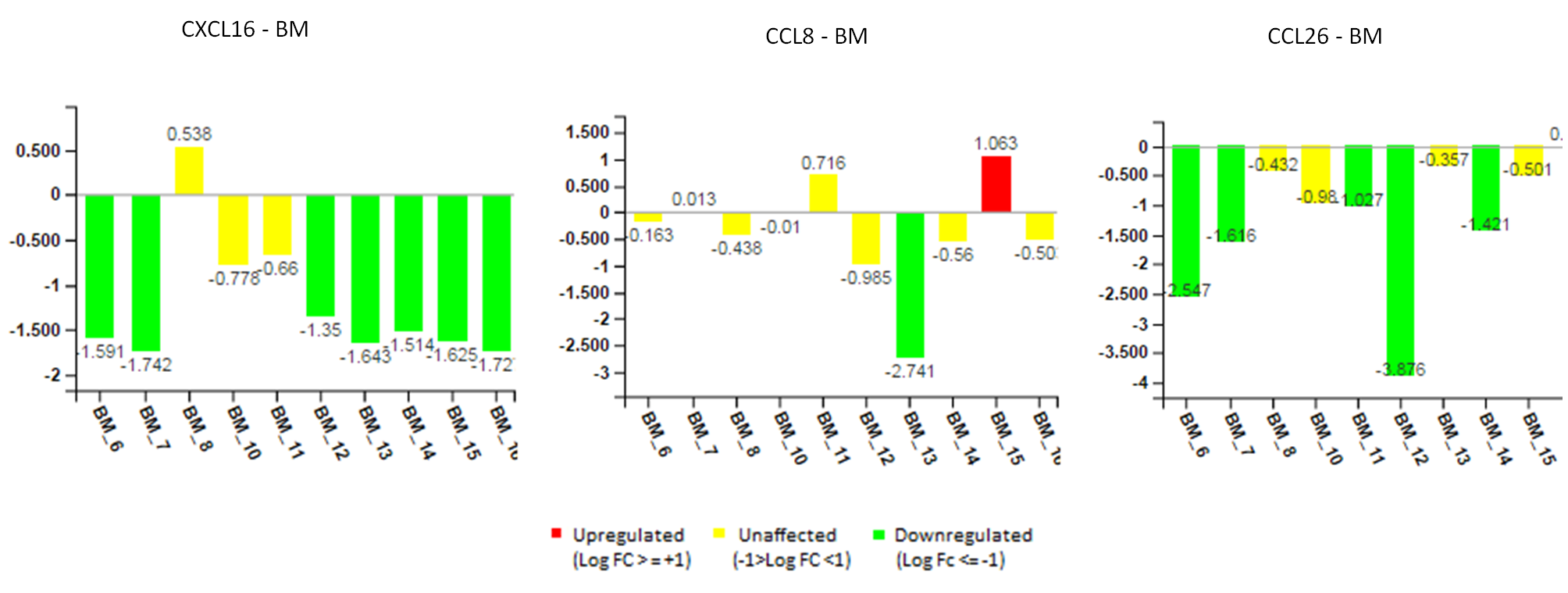

Supplement: Supplementary file 10 — Figure S7. Expression profile of genes activated by IL4 via JAK-STAT pathway. (TIFF 5004 kb) [file 12895_2018_70_MOESM10_ESM.tif]

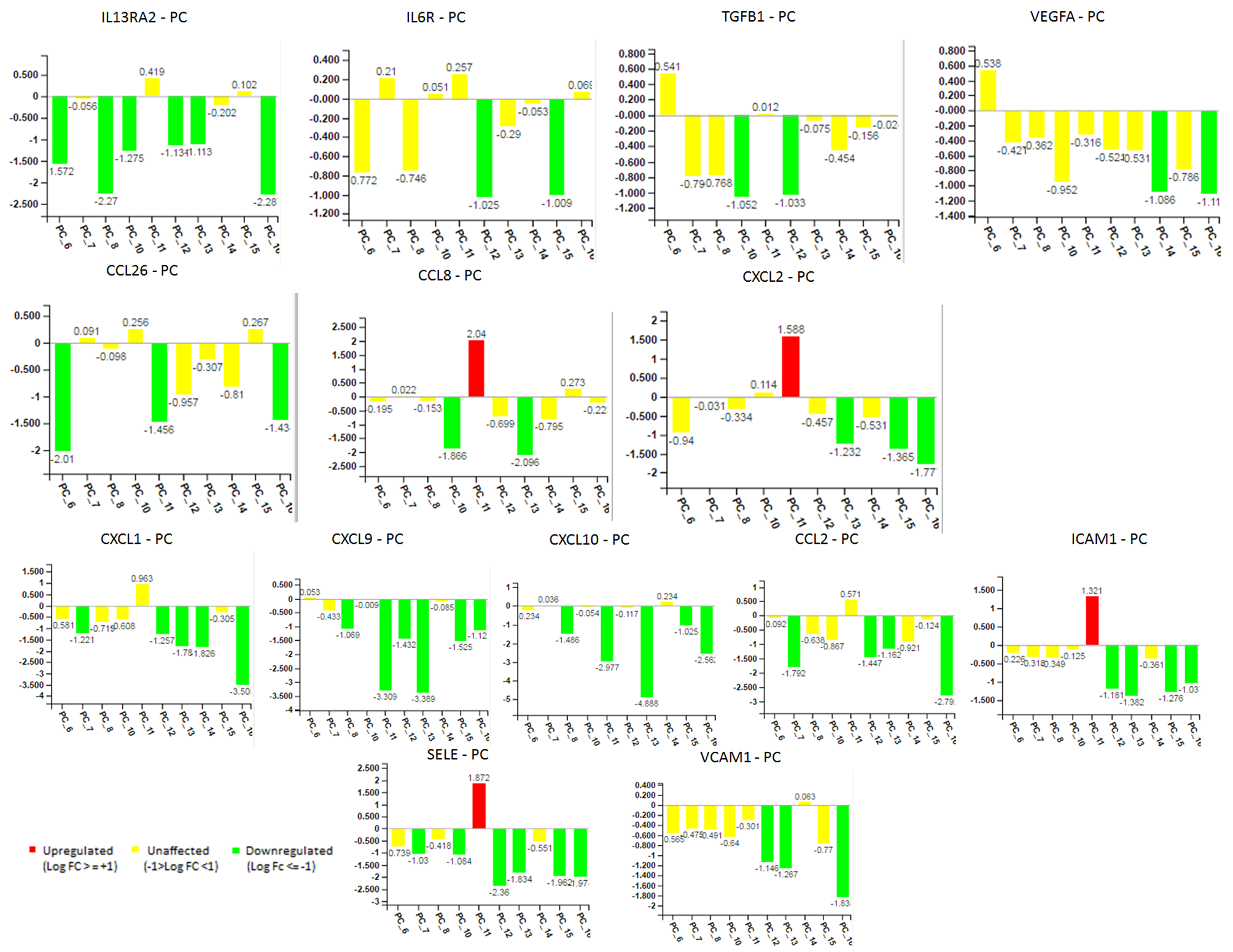

Supplement: Supplementary file 11 — Figure S8. Expression profile of inflammatory genes that show downregulation in PC samples. (TIFF 10440 kb) [file 12895_2018_70_MOESM11_ESM.tif]

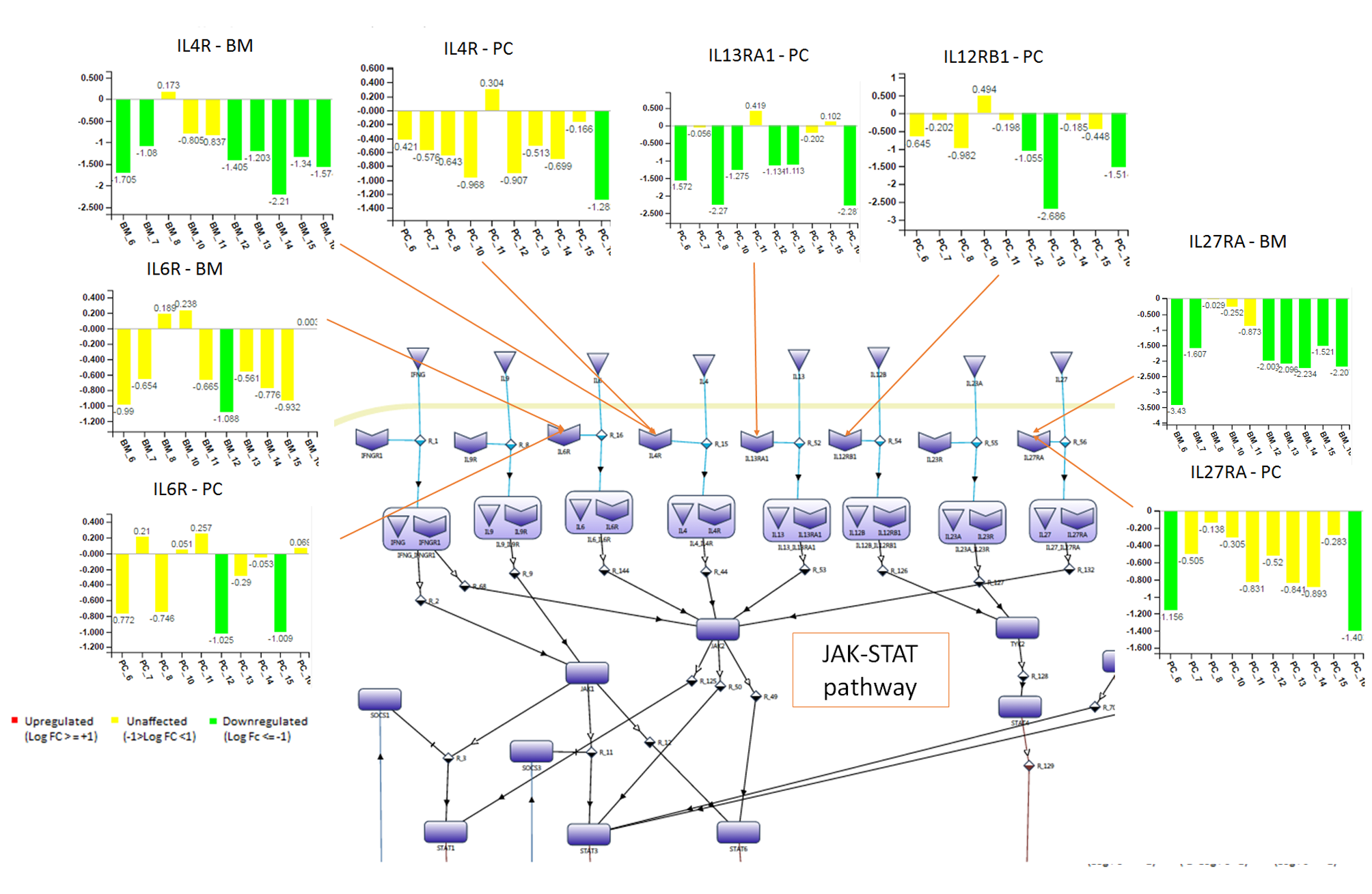

Supplement: Supplementary file 12 — Figure S9. Section of eSkIN Immune Response pathway showing various activators of JAK-STAT pathway and their expression profile in BM and PC samples. (TIFF 10437 kb) [file 12895_2018_70_MOESM12_ESM.tif]

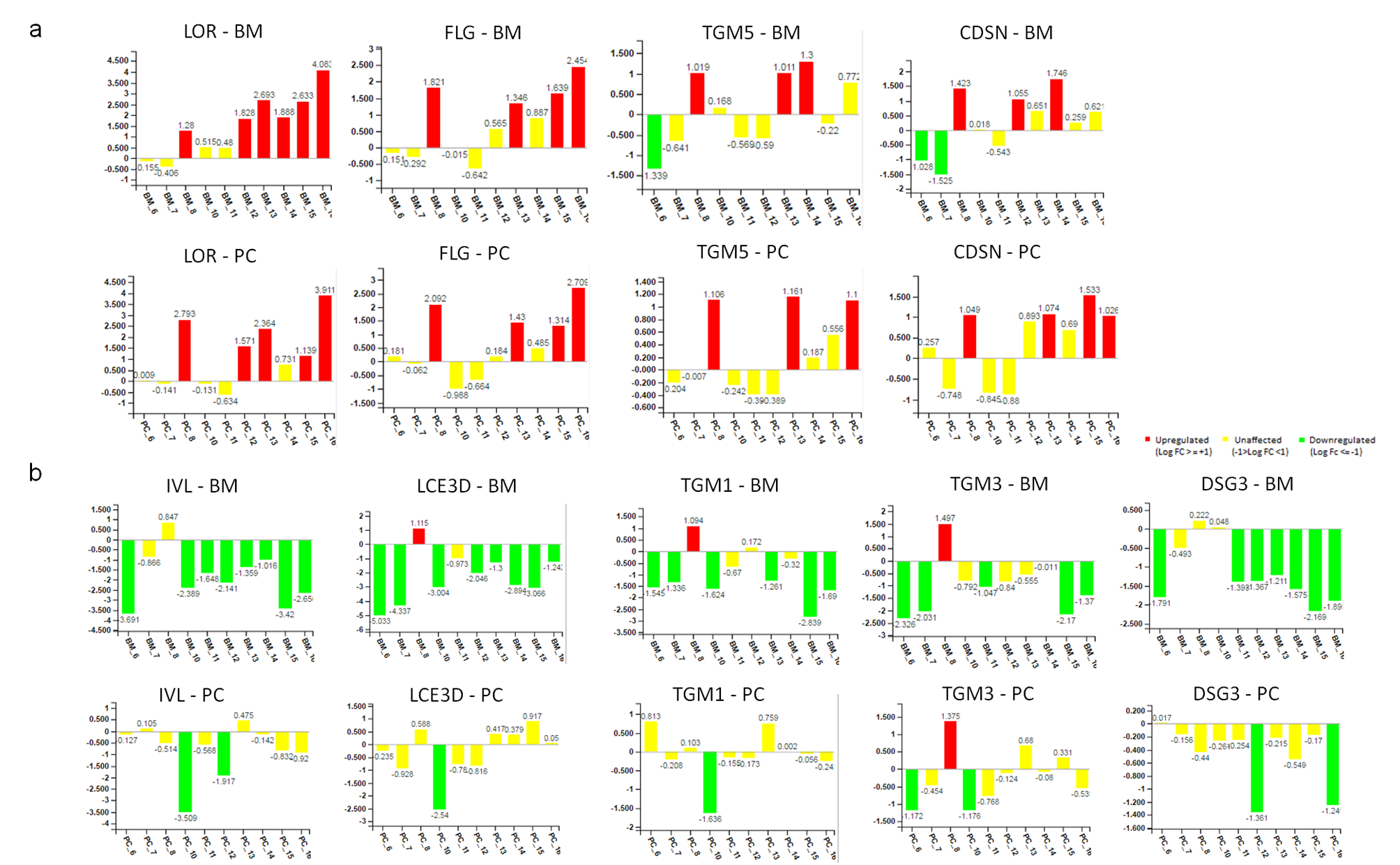

Supplement: Supplementary file 13 — Figure S10. Expression profile of skin barrier proteins in BM and PC samples. (a) Expression profile of genes that are upregulated in both BM and PC in order to restore barrier functions; (b) Expression profile of skin barrier genes that show treatment specific difference in their expressions. (TIFF 8378 kb) [file 12895_2018_70_MOESM13_ESM.tif]

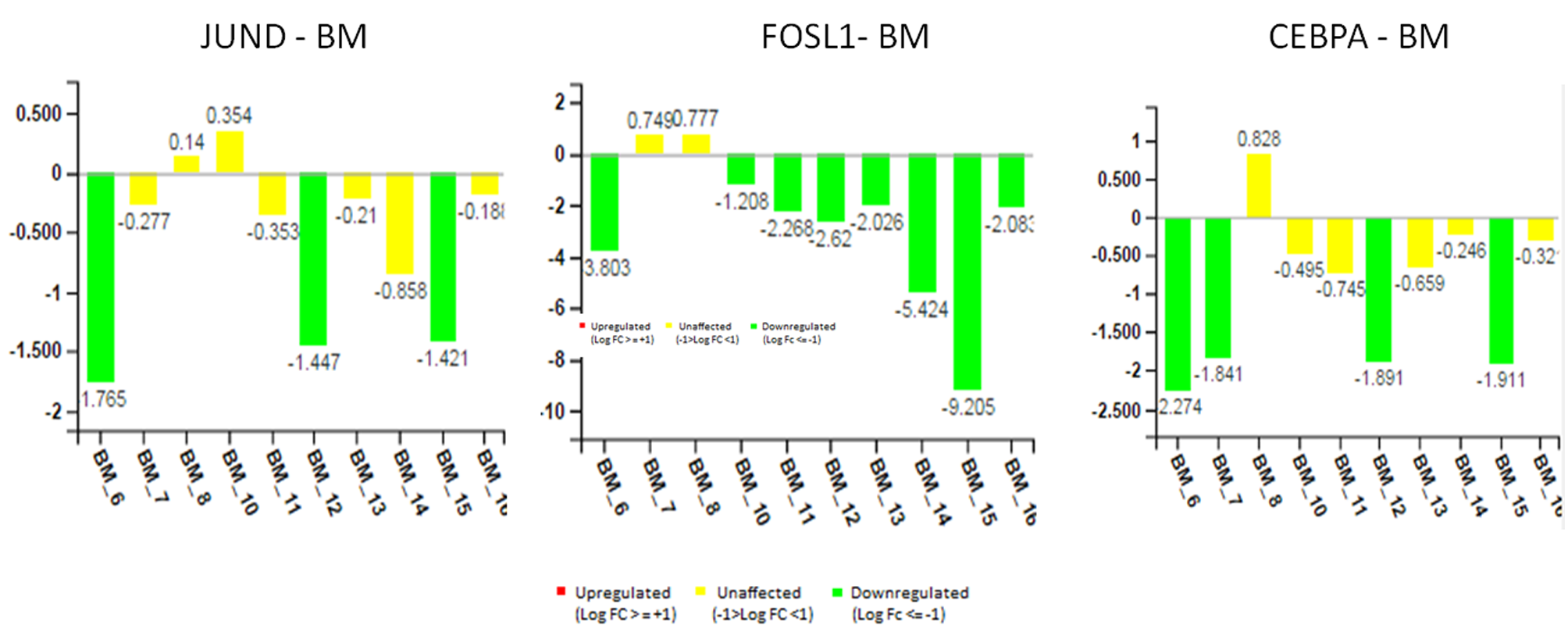

Supplement: Supplementary file 14 — Figure S11. Expression profile of important transcription factors of barrier proteins in BM. (TIFF 5538 kb) [file 12895_2018_70_MOESM14_ESM.tif]

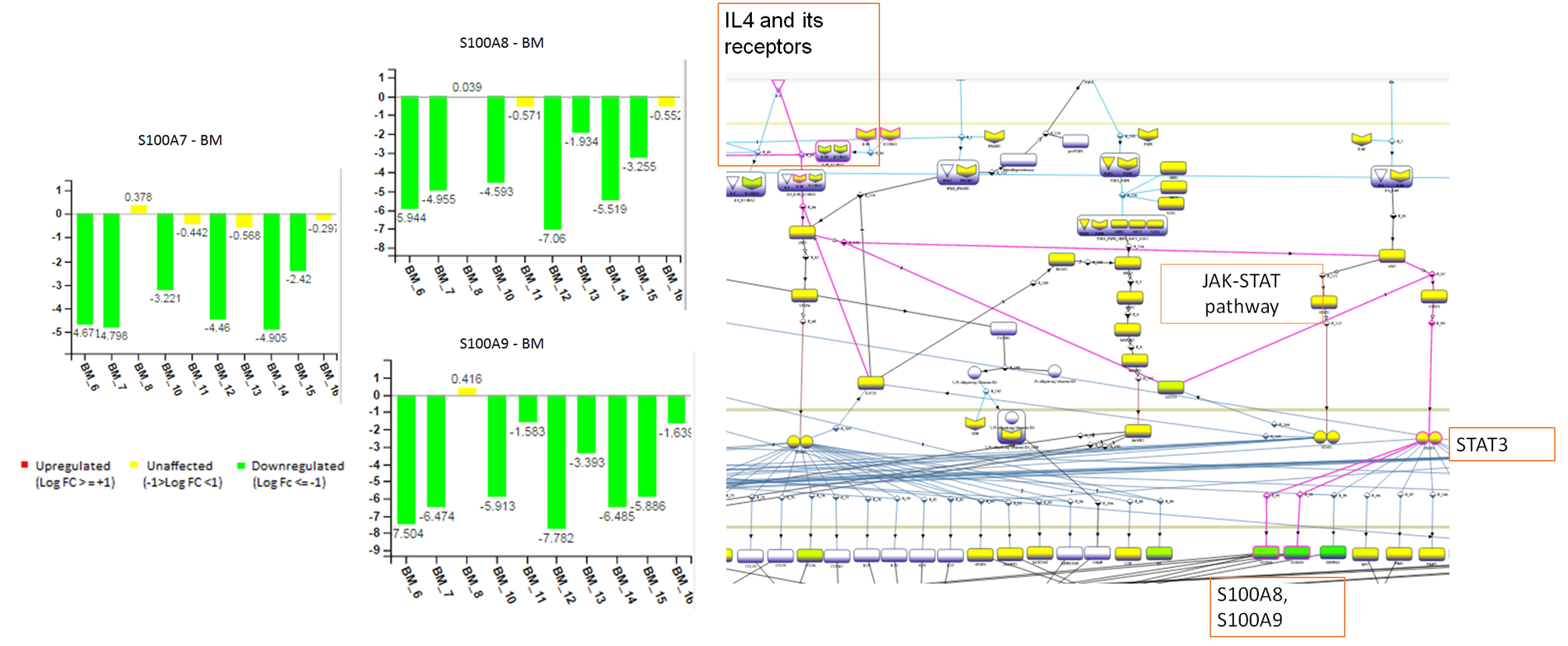

Supplement: Supplementary file 15 — Figure S12. Expression profile of anti-microbial peptides in BM samples and a section of eSkIN pathway showing their transcriptional regulation by IL4. (TIFF 5747 kb) [file 12895_2018_70_MOESM15_ESM.tif]

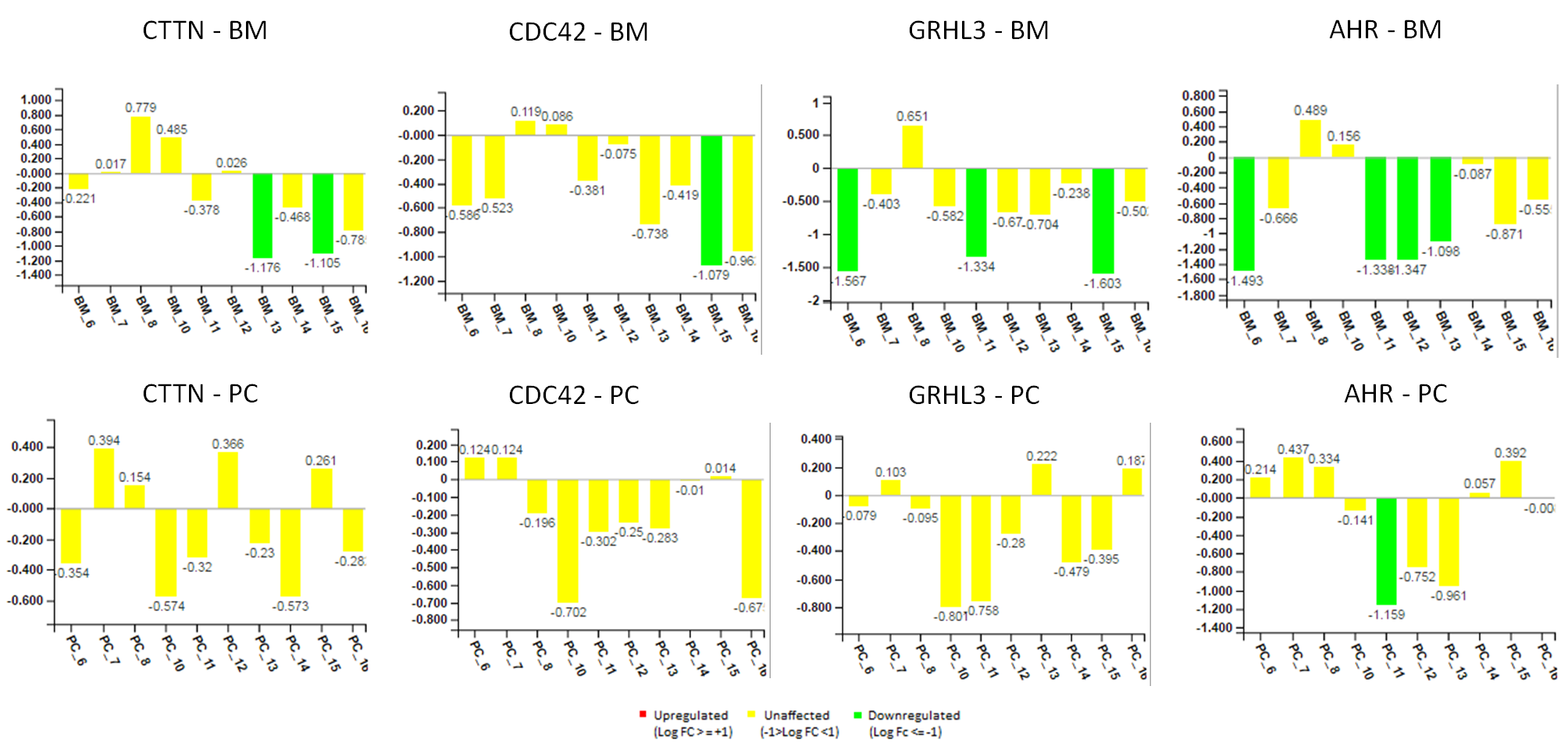

Supplement: Supplementary file 16 — Figure S13. Expression profile of junction proteins that show treatment specific difference in their expressions. (TIFF 6474 kb) [file 12895_2018_70_MOESM16_ESM.tif]

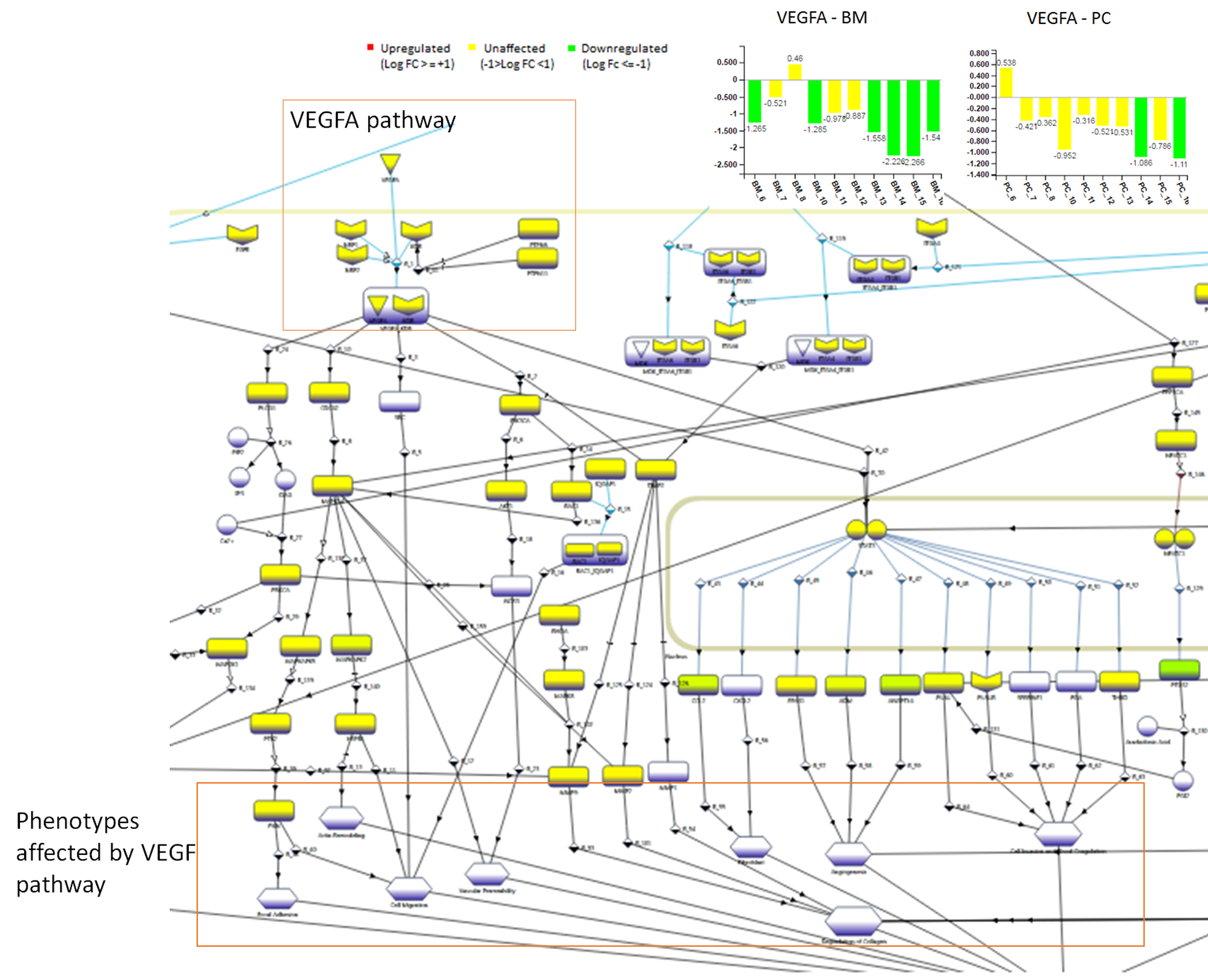

Supplement: Supplementary file 17 — Figure S14. Section of eSkIN Wound Healing pathway showing VEGF mediated activation of cellular functions like focal adhesion, actin remodeling, cell migration, vascular permeability, angiogenesis, degradation of collagen, cell invasion and blood coagulation, and expression profile of VEGF in BM and PC samples. (TIFF 12210 kb) [file 12895_2018_70_MOESM17_ESM.tif]

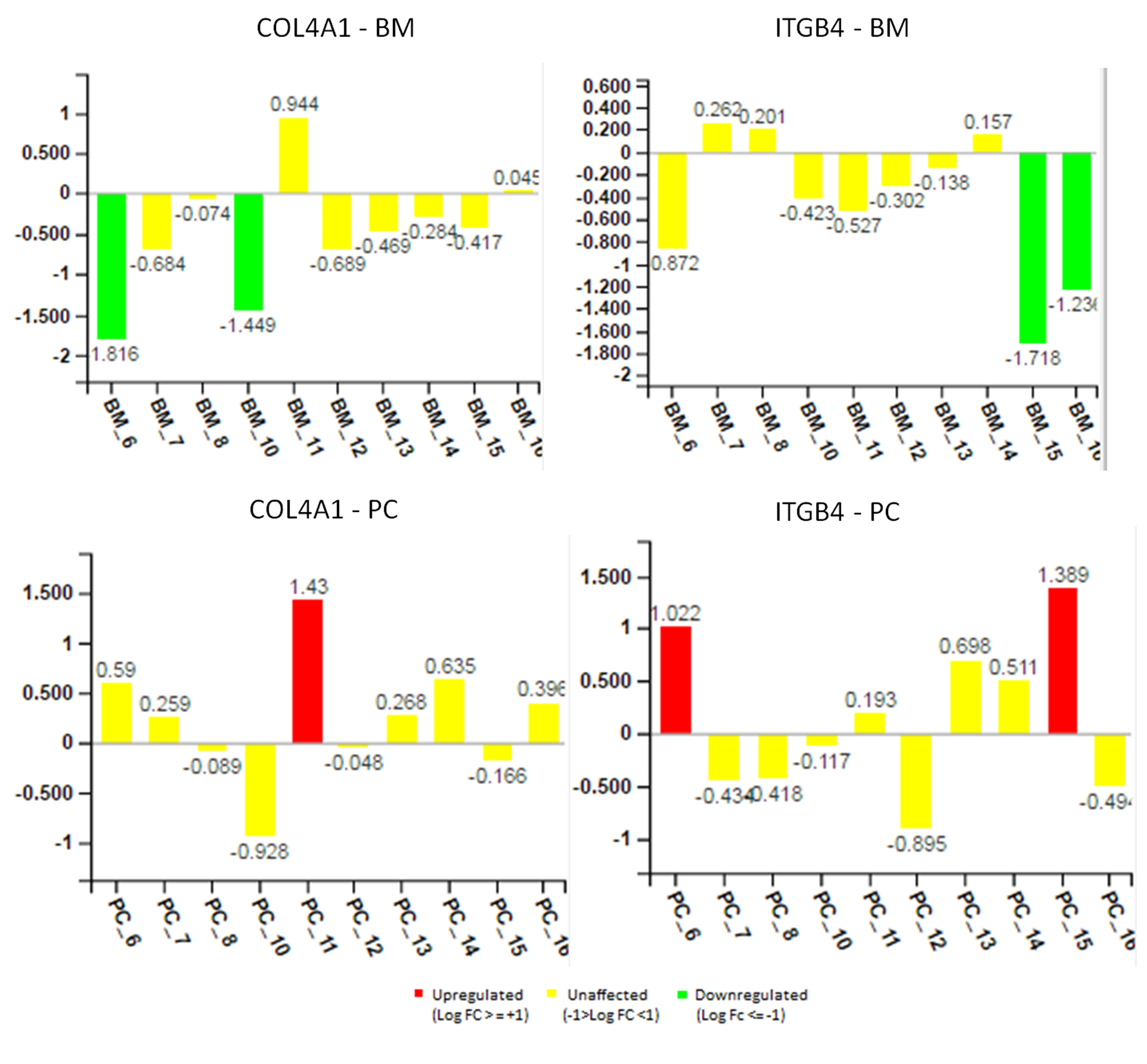

Supplement: Supplementary file 18 — Figure S15. Expression profile of basal layer genes that show treatment specific difference in their expressions. (TIFF 12463 kb) [file 12895_2018_70_MOESM18_ESM.tif]

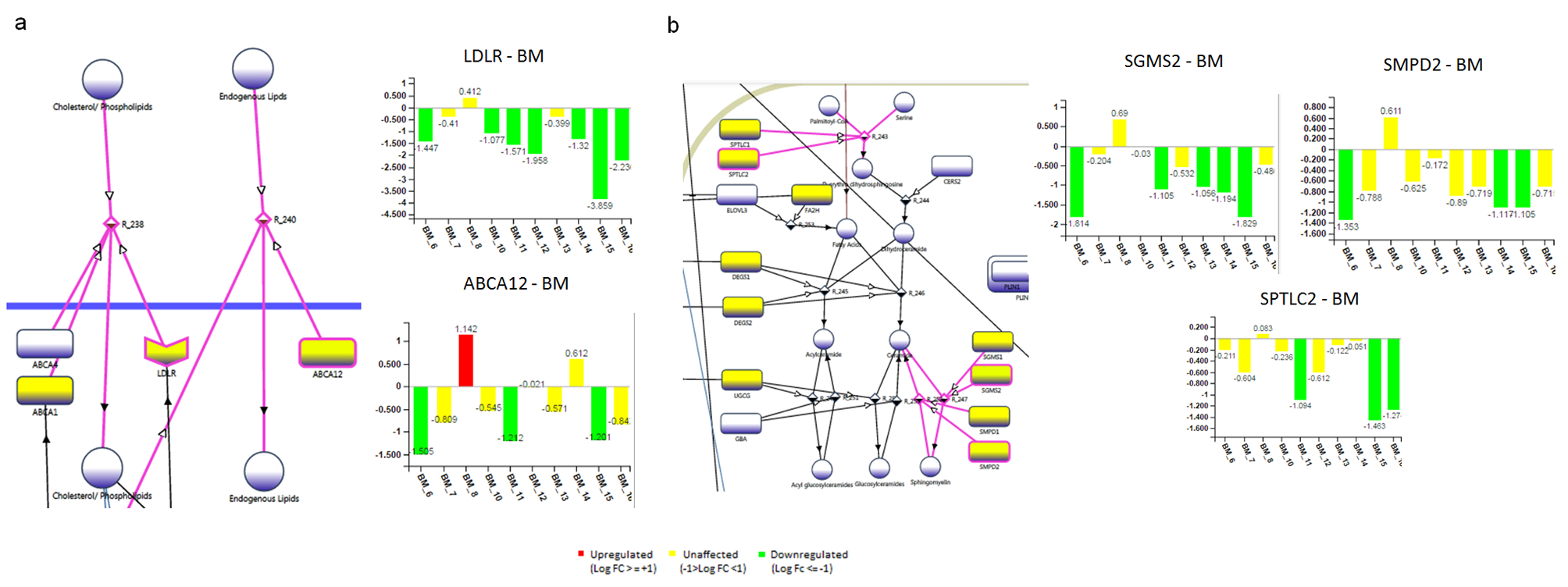

Supplement: Supplementary file 19 — Figure S16. Sections of eSkIN Lipid Synthesis pathway showing: (a) lipid transporters and their expression profiles in BM samples (b) enzymes involved in fatty acid conversion and their expression profiles in BM samples. (TIFF 5067 kb) [file 12895_2018_70_MOESM19_ESM.tif]

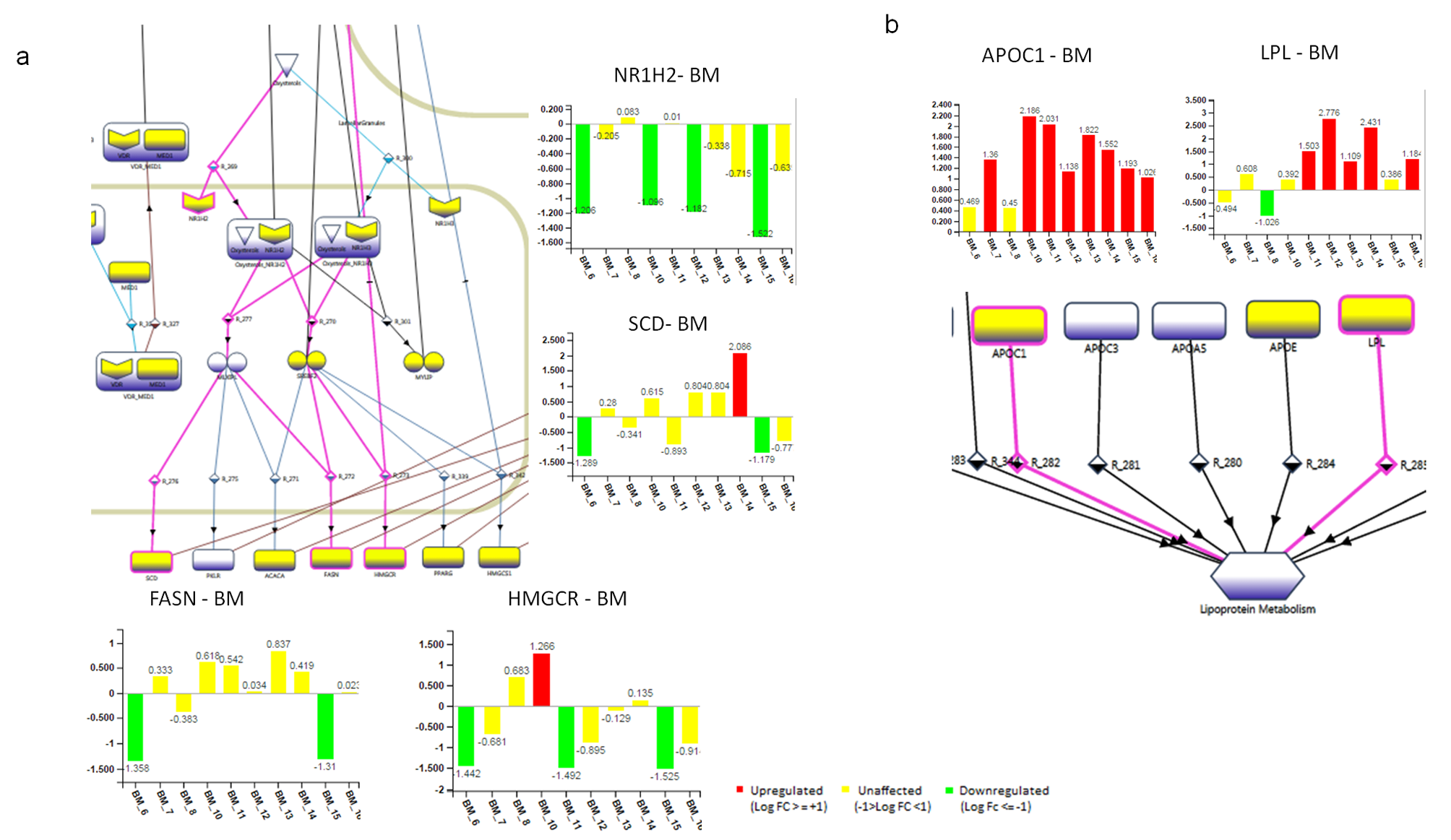

Supplement: Supplementary file 20 — Figure S17. Sections of eSkIN Lipid Synthesis pathway showing: (a) the genes involved in the synthesis of lipids and fatty acids and their expression profiles in BM samples (b) genes involved in lipid metabolism and their expression profiles in BM samples. (TIFF 7877 kb) [file 12895_2018_70_MOESM20_ESM.tif]

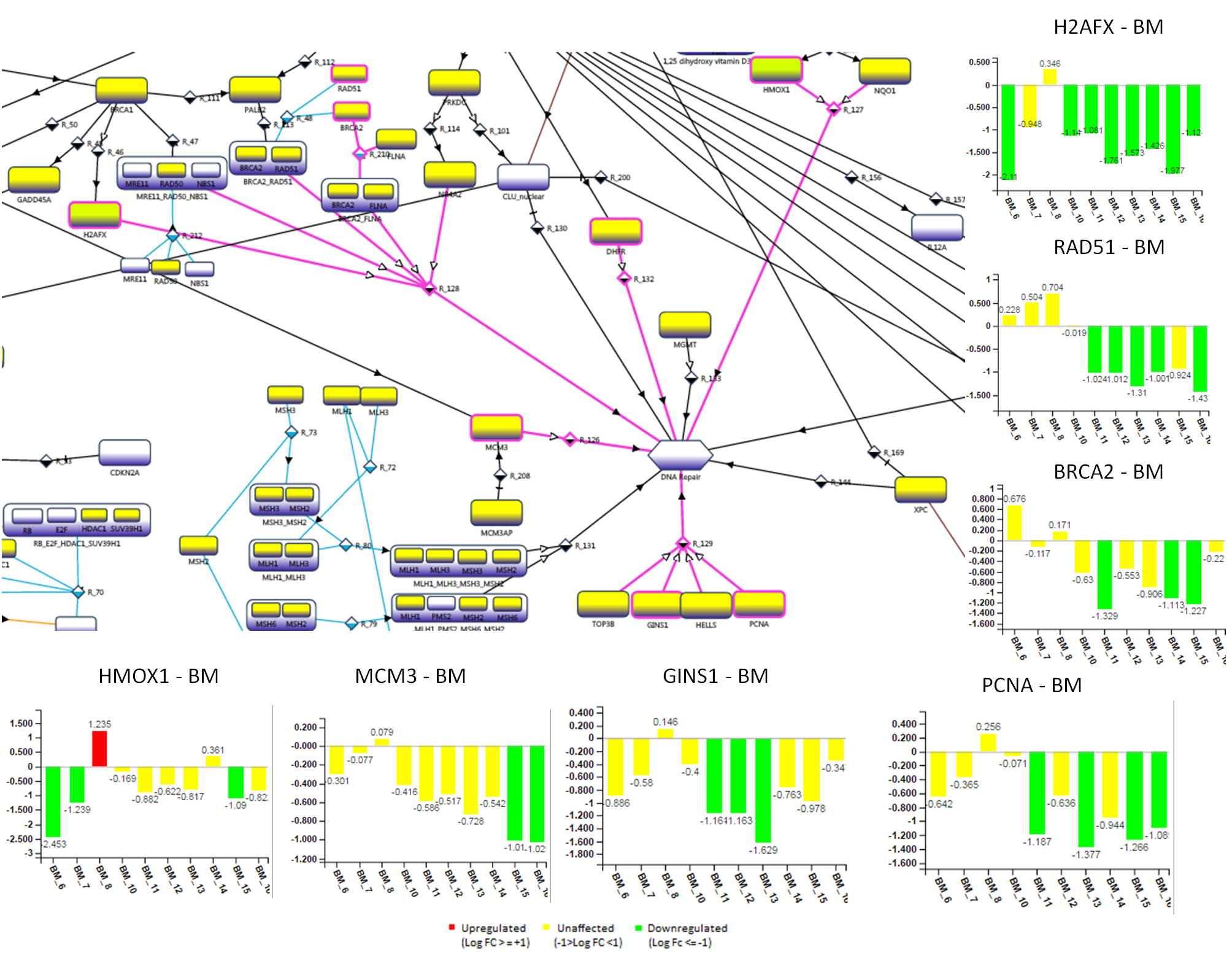

Supplement: Supplementary file 21 — Figure S18. Section of eSkIN DNA Damage and Repair pathway showing the genes involved in DNA repair mechanisms and their expression profiles in BM samples. (TIFF 10956 kb) [file 12895_2018_70_MOESM21_ESM.tif]
